# Co-occurring microflora and mucin drive *Pseudomonas aeruginosa* diversification and pathoadaptation

**DOI:** 10.1093/ismeco/ycae043

**Published:** 2024-03-28

**Authors:** Michael J Bottery, Helle Krogh Johansen, Jon W Pitchford, Ville-Petri Friman

**Affiliations:** Division of Evolution Infection and Genomics, School of Biological Sciences, University of Manchester, Manchester M13 9PL, United Kingdom; The Novo Nordisk Foundation Center for Biosustainability, Technical University of Denmark, 2800 Kongens Lyngby, Denmark; Department of Clinical Microbiology, Rigshospitalet, Copenhagen University Hospital, Copenhagen 9301, Denmark; Department of Clinical Medicine, Faculty of Health and Medical Sciences, University of Copenhagen, Copenhagen 2200, Denmark; Department of Biology, University of York, Wentworth Way, York YO10 5DD, United Kingdom; Department of Mathematics, University of York, Heslington, York YO10 5DD, United Kingdom; Department of Biology, University of York, Wentworth Way, York YO10 5DD, United Kingdom; Department of Microbiology, University of Helsinki, Helsinki 00014, Finland

**Keywords:** Pseudomonas aeruginosa, cystic fibrosis, polymicrobial infection, Stenotrophomonas maltophilia, Staphylococcus aureus, mucin, diversification, pathoadaptation

## Abstract

While several environmental factors contribute to the evolutionary diversification of the pathogenic bacterium *Pseudomonas aeruginosa* during cystic fibrosis lung infections, relatively little is known about the impact of the surrounding microbiota. By using *in vitro* experimental evolution*,* we show that the presence of *Stenotrophomonas maltophilia*, *Staphylococcus aureus,* or them both, prevent the evolution of loss of virulence, which repeatedly occurs in the absence of these species due to mutations in regulators of the *Pseudomonas* Quinolone Signal quorum sensing system, *vqsM* and *pqsR*. Moreover, the strength of the effect of co-occurring species is attenuated through changes in the physical environment by the addition of mucin, resulting in selection for phenotypes resembling those evolved in the absence of the co-occurring species. Together, our findings show that variation in mucosal environment and the surrounding polymicrobial environment can determine the evolutionary trajectory of *P. aeruginosa*, partly explaining its diversification and pathoadaptation from acute to chronic phenotype during cystic fibrosis lung infections.

## Introduction

*Pseudomonas aeruginosa* is an opportunistic pathogen that frequently colonizes the lungs of people with the genetic disorder cystic fibrosis (CF), causing severe, life-threatening infections. Although CF lung infections are often polymicrobial, *P. aeruginosa* is commonly considered to be the leading cause of irreversible lung tissue damage in people with CF (pwCF) [1]. Initial acute infections by *P. aeruginosa* are caused by strains most likely originating from environmental sources [2] and can be cleared to undetectable levels through targeted antibiotic regimens in approximately in half of the patients [3]. However, these initial acute infections often transition into long-term chronic infections during a process of genetic and phenotypic diversification, coupled with adaptation to the CF lung environment [4–6]. These chronic isolates frequently converge upon a specific pathoadapted phenotype [7, 8], characterized by a switch to an aggregative lifestyle (overproduction of alginate resulting in mucoid phenotype, increased biofilm production, and loss of motility) accompanied with alterations in metabolism (emergence of auxotrophs [9], increased metabolic reliance on amino acids and lactate, and reduced glucose metabolism [9]), increase in antibiotic resistance and reduction in growth rates [10]. In addition, chronic isolates often display a reduction in the production of quorum sensing-regulated virulence factors, which include pyocyanin, pyoverdine, elastase, and protease [11], indicative of reduced or loss of acute virulence [12]. Many possible environmental drivers have been proposed to explain this diversification [13–16] (reviewed in [2, 4, 5]), including the extensive use of antibiotics, the host immune system and nutrient availability in the lung. However, the role of interactions with surrounding microbes typical for polymicrobial CF lung infections [17] has received less attention even though they also likely shape the diversification of *P. aeruginosa* during CF lung infections [18–21].

Interspecies interactions with co-occurring bacteria could alter the rate, strength, and the trajectory of *P. aeruginosa* adaptation in CF lung. Theory predicts that antagonistic species interactions or abiotic conditions that reduce the focal species population densities should constrain its adaptation due to reduced mutation supply rate [22–24]. As *P. aeruginosa* population densities often fluctuate in time and can go through frequent bottlenecks, selection can favour the emergence of mutator genotypes, enabling quick accumulation of genetic diversity and rapid response to selection in CF lung [4, 25]. Moreover, co-occurring bacteria within the CF lung could alter the physiology and growth of *P. aeruginosa,* creating selection for *P. aeruginosa* diversification even in the absence of clear population density effects [21, 26]. For example, interactions with commonly coinfecting bacteria such as *Staphylococcus aureus* and *Stenotrophomonas maltophilia* can elicit changes in *P. aeruginosa* gene expression and virulence factor production [26–29]. These responses are partly driven by indirect competition for nitrogen, amino acids, and iron [30, 31], but also via direct interference competition mediated for example by antimicrobial secretions [32]. Competitive interactions could hence drive the evolution of niche specialism and character displacement between co-occurring species [33] potentially explaining changes in metabolism and the emergence of auxotrophs in CF lung [34]. Finally, some adaptations could be neutral in the selective environment but potentially beneficial or harmful when the environmental conditions change. For example, stress responses triggered by co-occurring species [35] and adaptation to synthetic cystic fibrosis media (SCFM) [13] have previously been shown to correlate with changes in antibiotic sensitivity in *P. aeruginosa*. Seemingly neutral evolutionary changes in one environment could thus have significant consequences for the success of antibiotic treatments depending on the direction of genetic trait correlations (trade-offs vs trade-ups [36]).

In addition to competition, co-occurring bacteria could provide benefits for *P. aeruginosa* via cross-feeding [37] or by providing protection for antibiotics [29, 38, 39]. Over longer evolutionary timescale, pairwise interactions with *S. maltophilia* have been shown to result in elevated rates of antibiotic resistance evolution [39], while mutations in lipopolysaccharide biosynthesis genes have been associated with increased fitness in the presence of *S. aureus* [20]. It remains however unclear if different types of bacterial species interactions result in phenotypic convergence of lung-adapted *P. aeruginosa*, or if distinct interspecies interactions select for specialized and locally adapted *P. aeruginosa* lineages within the CF lung. While there is evidence for convergent evolution towards generalized adaptations in CF lung, such as reduced growth rate and antibiotic resistance [7], high levels of between- and within-patient diversity exists. This suggests that the benefit of specialized adaptations, such as maintenance of virulence factor production [40, 41], may depend upon the specific composition of the co-occurring bacteria in the lung [7]. Importantly, the presence of co-occurring species such as *S. maltophilia* is a strong predictor for decreased lung function due to the increased probability of secondary *P. aeruginosa* infection [42]. This suggests that interspecies interactions play a role in the outset of early acute infection when the selection for *P. aeruginosa* diversification is considered to be at its fastest [7].

The selection imposed on *P. aeruginosa* by other CF bacteria could also be sensitive to environmental factors such as presence of mucins, which are high molecular weight glycoproteins that form a protective layer throughout the airways [43]. The dysregulation of mucin production in CF airways contributes to the high viscosity and spatial heterogeneity of CF sputum [44] and can alter the dispersal and virulence factor production of *P. aeruginosa* [13, 45] by altering the expression of *P. aeruginosa* virulence factors, triggering the down regulation of quorum sensing genes, siderophore production and toxin secretion [45]. Each of these factors influences the ability of *P. aeruginosa* to interact with other co-occurring bacterial species [46, 47]. While recent studies have established that mucin alone can both regulate virulence factor production [45] and affect the evolutionary diversification of *P. aeruginosa* [13, 48], it is less clear if mucin changes the strength of species interactions, potentially altering *P. aeruginosa* adaptation in polymicrobial CF communities.

Here, we use experimental evolution to test *in vitro* if the evolutionary trajectory of *P. aeruginosa* is dependent upon the identity of the commonly co-occurring bacterial species *S. aureus* or *S. maltophilia*, in pairwise and three-species co-cultures in the presence and absence of mucin. We hypothesized that co-occurring species will determine the evolutionary trajectory of *P. aeruginosa*, selecting for repeatable adaptations that are unique to co-occurring species identities. The presence of mucin could further shape adaptation by allowing additional niche dimensions (e.g., spatial or nutritional) or by affecting the strength of interspecies interactions. To test these hypotheses, *P. aeruginosa* was evolved in synthetic cystic fibrosis media (SCFM) in the presence (+mucin) or absence (−mucin) of mucin in the absence of co-occurring species ([None]) or in two pairwise (*S. maltophilia* [SM] or *S. aureus* [SA]) and one three-species ([SM + SA]) community for ~130 generations (Fig. S1). At the end of the experiment, we measured changes in the phenotypic traits commonly associated with pathoadaptation (growth dynamics, secreted products, adherence, and antibiotic resistance) and used deep sequencing of evolved populations to assess the impact on within- and between-population adaptation and diversification in *P. aeruginosa*. We found that co-occurring species identity determined *P. aeruginosa* phenotypic diversification and adaptation in the absence of mucin, and that selection in three-species communities led to a separate evolutionary trajectory. Crucially, selection by co-occurring species maintained virulence of *P. aeruginosa*, while the lack of other species, or the presence of mucin, selected for mutations in virulence genes, including nonsynonymous mutations in in the regulators of the Pseudomonas Quinolone Signal quorum sensing system, *pqsR* and *vqsM*. Together, our findings show how variation in both mucosal environment and composition of the co-occurring bacteria of the lung could drive the evolutionary trajectory of *P. aeruginosa*, potentially explaining the transition from acute to chronic phenotype during CF airway infections.

## Methods

### Strains, culture conditions, and evolution experiment

*P. aeruginosa* PAO1 chromosomally tagged with dTomato via mini-Tn7 [38] was used as the founder of the evolution experiment. *S. maltophilia* (SM518651) and *S. aureus* (SA521307) isolated from CF sputum samples provided by Prof. Helle Krogh Johansen, Rigshospitalet, Copenhagen [38] were used as co-occurring species in the evolution experiment. The evolution experiment and subsequent culturing of evolved isolates for phenotyping was conducted with Synthetic Cystic Fibrosis Media (SCFM), prepared following the protocol outlined in [49] with the addition of thiamine (1 mg/L), nicotinic acid (1.2 mg/L), calcium pantothenate (0.25 mg/L), and biotin (0.005 mg/L) to support the growth of *S. aureus* [50]. Evolution treatments containing mucin were supplemented with 5 g/L Type II porcine stomach mucin which contains MUC5AC. The same batch of mucin was used throughout the evolution experiment to control for any variation in media composition. Although porcine mucin is a relative crude formulation and may have contained a mixture of glycoproteins and other compounds, it was readily accessible for a highly replicated and factorially designed evolution experiment, and has previously been used in several previous experiments [13, 51, 52]. Mucin was prepared following the protocol outlines in Turner *et al.* [53], briefly 250 mg of mucin was sterilized with UV for 4 hours with mixing every 30 mins. Sterilized mucin was added to the SCFM buffered based solution 24 hours prior to use. A total of 5 mg/L mucin has previously been shown to promote the formation of self-aggregating biofilm structures and population divergence in *P. aeruginosa* [51, 54]. SCFM supported the growth of both *S. aureus* and *S. maltophilia*, and growth was not significantly impacted by the addition of mucin (Fig. S15). Mueller–Hinton agar was used for antibiotic susceptibility testing of evolved isolates. Overnight cultures of *P. aeruginosa* PAO1 grown at 37°C shaken at 180 rpm were plated out onto LB agar to isolate single colonies. Each evolution line was founded using an individual colony. The evolution experiment was factorially designed to incorporate four community treatments, no co-occurring species (None), *S. aureus* (SA), *S. maltophilia* (SM) and *S. maltophilia* plus *S. aureus* (SM + SA), and two mucin treatments, mucin absent (−mucin) and mucin present (+mucin), with six independent populations for each of the eight treatments for a total of 48 evolving populations. The evolution experiment was performed using 1.5 ml cultures in 24-well plates incubated without shaking at 37°C. Populations were propagated by transferring 1% of the population into fresh media every 24 hours. In treatments with the presence of co-occurring species, fresh co-occurring species were pre-inoculated into the fresh media 6 hours prior to the transfer to prevent them from going extinct. This resulted in an average pre-transfer density of 3.2 × 10^8^ for *S. aureus* and 1. 2 × 10^8^ CFU for *S. maltophilia* (Fig. S3). Surviving *S. aureus*, or *S. maltophilia* were not separated from *P. aeruginosa* upon transfer**.** The average densities of surviving *S. aureus* and *S. maltophilia* at the end of each transfer were 1.61 × 10^5^ CFU/ml and 1.09 × 10^6^ CFU/ml, respectively, and 1% of these populations were transferred. On average, in 99.98% of the *S. maltophilia* population and 99.999% of the *S. aureus* populations being refreshed with unevolved ancestral strains at the start of each serial transfer cycle. The densities of *P. aeruginosa* were monitored daily by plating onto Pseudomonas Selective Agar, and the density of the co-occurring species was measured every 5 transfers through plating onto Mannitol Salt Agar for *S. aureus* or Stenotrophomonas Selective Media (low salt LB agar (NaCl 0.5 g/L) containing 32 μg/ml imipenem and 5 μg/ml vancomycin) for *S. maltophilia* (Fig. S1). Mean pre-transfer densities of *P. aeruginosa* were between 1.68 × 10^9^ and 5.63 × 10^9^ CFU/ml, accounting for over 98.7% of the population (Table S6). Populations were propagated for 20 transfers, equating to approximately 130 *P. aeruginosa* generations (~6.64 generations per day). Following evolution at transfer 20, 12 randomly selected *P. aeruginosa* colonies were isolated from each population, resuspended in sterile PBS and frozen in replicate 20 μl aliquots at −80°C in 25% glycerol for phenotyping. Whole populations were frozen every five transfers in 25% glycerol at −80°C.

### Phenotyping

For each phenotypic measurement (pyoverdine, pyocyanin, protease, elastase, adherence, and growth dynamics) aliquots of each of the 576 evolved isolates, together with 12 replicates of the ancestral strain, were defrosted and diluted 1:100 in SCFM. These were then used to inoculate three independent cultures per isolate in SCFM minus mucin and grown for 24 hours at 37°C with no shaking in 96-well plates prior to phenotyping.

#### Pyoverdine

The fluorescence of overnight cultures was measured at 460 nm following excitation at 400 nm, using a Tecan Infinite M200 Pro microplate reader. The gain was kept constant across all 96-well plates to allow comparison across independent measures. Optical Density (OD) was measured at 600 nm and the per capita pyoverdine production was calculated as relative fluorescence intensity/OD_600_.

#### Pyocyanin

The cell density of overnight cultures was measured at OD_600_, followed by centrifugation at 4000 rpm for 10 mins. A total of 100 μl of the supernatant was then transferred into fresh 96-well plates and OD at 695 nm was recorded using a Tecan Sunrise microplate reader. Per capita pyocyanin production was calculated as OD_695_/OD_600_ of pre-centrifuged cultures.

#### Protease

The cell density of overnight cultures was measured at OD_600_, followed by centrifugation at 4000 rpm for 10 mins. A total of 75 μl of the supernatant was then transferred into 125 μl Azocasein Solution (2% Azocasein, 2 mM CaCl_2_, 40 mM tris–HCl, pH 7.8), mixed thoroughly by pipetting and incubated for 45 mins at 37°C. Following incubation, 600 μl 10% trichloroacetic acid was added, the solution was mixed by pipetting and incubated at room temperature for 15 mins. Solutions were centrifuge for 10 mins at 4000 rpm to remove precipitates and 120 μl of the supernatant was added to 100 μl 1 M NaOH and OD_440_ was measured.

#### Elastase

The cell density of overnight cultures was measured at OD_600_, followed by centrifugation at 4000 rpm for 10 mins. A total of 20 μl of the supernatant was then transferred into 180 μl Elastin Congo-Red buffer (10 mg/ml Elastin Congo-Red, 1 mM CaCl_2_, 100 mM Tris, pH 7.5, sterile filtered) in 96-well plates and incubated at 37°C for 4 hours with shaking at 400 rpm, orbital radius of 3 mm. Plates were then centrifuged at 4000 rpm for 10 mins, 100 μl of the supernatant was removed and OD_495_ was measured. Per capita elastase production was calculated as OD_495_/OD_600_ of pre-centrifuged cultures.

#### Adherence

Adherence was measured as the ability to bind to NUNC peg-lids during growth following [7]. A total of 150 μl cultures of each isolate were grown in SCFM for 24 hours in NUNC 96-well plates (Cat no. 167008) with NUNC peg lids (Cat no. 445497) at 37°C with no shaking. Following growth, the density of the cultures was measured using OD_600_. The peg lids were removed and washed in sterile PBS to remove non-adhering cells. The washed peg lids were transferred to fresh microtiter plates containing 160 μl 0.01% crystal violet and left to stain for 15 mins at room temperature. The peg lids were then washed three times in fresh 180 μl sterile PBS to remove unbound crystal violet. Stained peg lids were transferred to fresh microtiter plates containing 180 μl of 99% ethanol to dissolve the bound crystal violet. Dissolved crystal violet was quantified by measuring OD_590_ and adherence was normalized to the initial cell density to calculate per capita adherence, OD_590_/OD_600_.

#### Growth dynamics

Cultures were incubated in SCFM for 6 hours at 37°C to allow cells to reach mid-exponential phase, and then diluted 1 in 100 into fresh SCFM at a final volume of 200 μl in NUNC Microplate Edge 2; 96 flat bottom plates. The outer reservoir was filled with sterile water to prevent evaporation from the wells and the plate was incubated for 24 hours at 37°C in a BMG SPECTROstar Nano microplate reader. OD_600_ was measured every 20 mins. Growth rates were calculated as the maximum slope of the log_2_ transformed OD_600_ covering four time points (1 hour 20 mins growth period), lag phage was calculated to end when growth rate reached 10% of the maximum achieved growth rate and maximum OD of each culture was recorded.

#### Antibiotic sensitivity

As changes in antibiotic sensitivity have previously been described in response to interspecies stress [35] MICs for ciprofloxacin (concentration range 0.002–32 μg/ml), tobramycin (0.016–256 μg/ml), meropenem (0.002–32 μg/ml), aztreonam (0.016–256 μg/ml), and colistin (0.016–256 μg/ml) were determined using E-TEST strips (bioMerieux UK Ltd). Colonies of each isolate tested (minimum of 6 randomly selected colonies per treatment) were resuspended in PBS at a density of 0.5 McFarland Standard. Suspensions were spread onto 120 × 120 mm square Mueller–Hinton plates and allowed to completely dry. E-TEST strips were placed on to the plates following the manufactures instructions and the plates were incubated at 37°C for 20 hours after which the MICs were recorded.

### Phenotypic MicroArray

Within host evolution during chronic infection can lead to changes in metabolism and the emergence of auxotrophs [34]. To determine the metabolic profiles of selected evolved isolates Phenotypic MircoArrays (Biolog) were conducted. Five colonies isolated from five different randomly selected populations evolved in the presence of mucin from each treatment were tested on three different Phenotypic MircoArray (PM) plates, PM1, PM2 and PM3. PM1 and PM2 contain 190 different sole carbon sources, and PM3 contains 95 different sole nitrogen sources. In addition, the ancestral PAO1 isolate was measured in triplicate. The standard Phenotypic MircoArray for gram negative bacteria was followed. Each strain was streaked out for single colonies on SCFM agar plates, and a single colony was suspended in inoculating fluid (IF-0 GN/GP, Biolog) to a transmittance of 42%. This was then diluted 1:5 with inoculating fluid containing Biolog Redox Dye A to create a final transmittance of 85%. A total of 100 ul of this was used to inoculate each well of PM1 and PM2 plates. For PM3 sodium succinate/ferric citrate solution was added to the inoculum at a final concentration of 20 mM sodium succinate/2 μM ferric citrate. A total of 100 ul of this mixture was used to inoculate each well of PM3 plate. The plates were incubated for 24 hours at 37°C and redox colour change was measured as OD590 – OD750.

### Genome sequencing and analysis

Whole genomes of the ancestral *P. aeruginosa* (PAO1::dTomato), *S. maltophilia* (SM518651) and *S. aureus* (SA521307) were extracted from monocultures using DNeasy Blood and Tissue extraction kits (Qiagen). Total DNA was sequenced by MicrobesNG (http://www.microbesng.uk). Ancestral *P. aeruginosa* reads were mapped to the *P. aeruginosa* PAO1 genome reference (GenBank accession AE004091.2) using BWA-MEM to determine the initial genotype of the ancestral strain. Single-nucleotide variants and small indel events were detected using GATK UnifiedGenotyper and annotated using SnpEff. Mutations present in the ancestral clone were excluded from the analysis of the evolved isolates, resulting in a set of mutations that were acquired during the selection experiment. *S. maltophilia* and *S. aureus* genomes were assembled de novo using SPAdes version 3.7 [55]. A single isolate from each of the evolved populations was randomly selected for sequencing. Isolates were sub-cultured in SCFM media and total DNA extracted and sequenced by MicrobesNG. Variants were called using BreSeq [56] Consensus Mode for clonal samples.

Each of the evolved populations at the end of the experiment at transfer 20 were sequenced. Each of the frozen evolved populations were thawed and 500 ul removed for DNA extraction. Total genomic DNA was extracted using the DNeasy Blood and Tissue extraction kit (Qiagen) according to the manufacturer’s instructions. The integrity of the DNA was assessed on a 0.75% agarose gel, and concentration estimated by Qubit dsDNA BR Assay Kit (Thermo Fisher Scientific). Library preparation and sequenced of total genomic DNA was conducted by Earlham Institute (http://www.earlham.ac.uk), using Nextera DNA Flex library preparation protocol and 150 base pair paired-end reads on the Illumnia NovaSeq 6000 platform. Read quality was assessed using FastQC, sequence adapters were trimmed using Trimmomatic [57] and quality assessed using Samtools [58], BedTools [59] and bwa-mem [60]. Trimmed reads were mapped to a combined reference containing PAO1 (AE004091.2) and the *de-novo* assembly of the *S. maltophilia* and *S. aureus* strains. Variants were called using BreSeq [56] Polymorphism Mode for population samples. The mean read depth was 379 reads for the PAO1 reference, 41 reads for the *S. maltophilia* contigs and 7 reads for the *S. aureus* contigs. The level of detection for variants in the evolved PAO1 populations was 5%. To produce a set on mutations accumulated throughout the evolution experiment, variants that were also present in the ancestral strain were excluded. In total we observed 489 mutations in the PAO1 populations across 48 population (Figs S11 and S12). The read depth was insufficient to analyse variants within the *S. maltophilia* and *S. aureus* populations.

### Phenotyping transposon mutants

To determine the effect of loss of function of the most frequently mutated genes in the evolved isolates the phenotypes of transposon mutations of each gene were measured. Mutant strains (PW2812, pqsR::ISlacZ/hah; PW3282, PA1264::ISphoA/hah; PW3811, aer::ISlacZ/hah; PW4287, PA1874::ISlacZ/hah; PW4792, vqsM::ISlacZ/hah; PW8253, tadZ::ISphoA/hah) were obtained from the *P. aeruginosa* PAO1 transposon mutant Library [61]. The location of the transposon insertion was confirmed by PCR using the suggested PCR primers flanking the insertion site and transposon-specific primer (ISphoA/hah 5’cgggtgcagtaatatcgccct-3′, ISlacZ/haha 5′-gggtaacgccagggttttcc-3′) paired with a flanking primer for 10 separate colonies from each mutant stock. The parental PAO1 strain, MPAO1, was also obtained as used a wildtype control during phenotyping. Measurements of pyoverdine, pyocyanin, protease, and elastase production, adherence and growth dynamics were conducted as described for the evolved isolates. 12 independent biological replicates of each transposon mutant and the parental strain, MPAO1, were conducted.

### Statistical analysis

**Figure 1:**
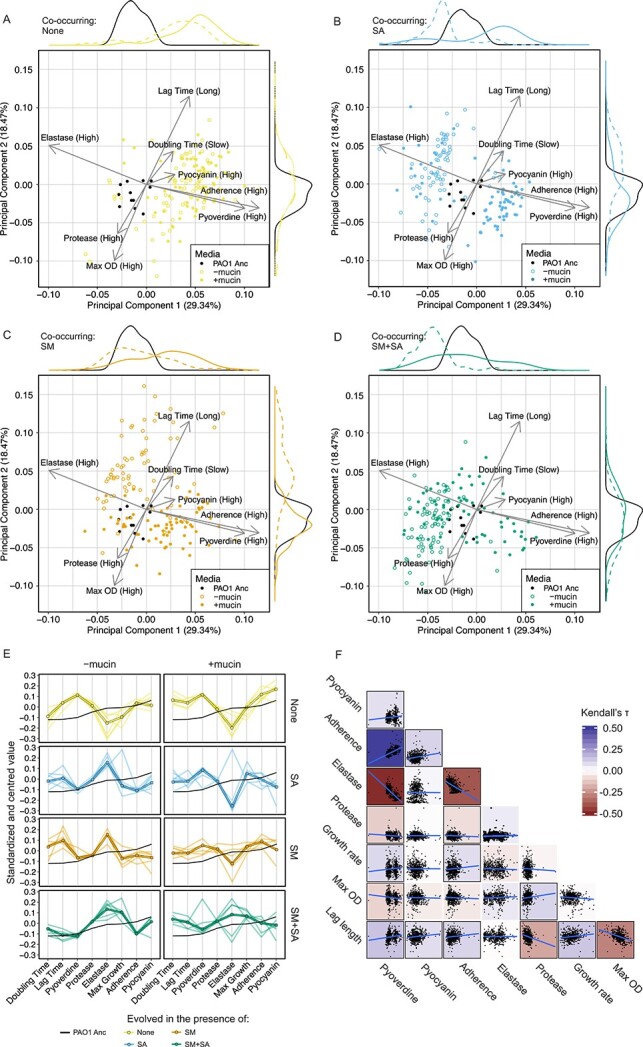
Phenotypic adaptation of *P. aeruginosa* depends on both the microbial community composition and on the presence of mucin. (A-D) Ordination plots from a principal component analysis of phenotypic trait measurements of ancestral strain (black points, n = 12) and evolved isolates (n = 72 per treatment *S. maltophilia* [SM], *S. aureus* [SA], *S. maltophilia* plus *S. aureus* [SM + SA]), produced on the complete set of evolved isolates from all treatments. The first two components describe 48% of the variance. The first three components accounted for 61% of the variance, PC3 shown in Fig. S9. The overlayed loadings plot (grey arrows) shows the extent to which each phenotypic trait contributes to each principal component and the separation of the points. Points represent individual isolates, the density of points across each axis is shown at the top (PC1) and right (PC2) of the plots. Panels facetted by treatment, with open points representing isolates from the absence of mucin and filled points from the presence of mucin. (E) Parallel coordinate plot of standardized phenotypic trait measurements. Bold line shows the median phenotype across all independent populations (n = 72), individual light lines show the median phenotype within populations (n = 12). The black line shows the median phenotype of the ancestral strain (n = 12). (F) Correlation matrix showing correlation between phenotypic measures across all evolved isolates and treatments. Correlation calculated using Kendall rank correlation coefficient with shading showing the Kendall’s τ coefficient value. A black box around the shaded area shows correlation is significant (Kendall’s τ *P* < 0.05). Raw phenotypic data is presented in Fig. S4.

All statistical analysis was conducted in R (version 3.3.3). Assumptions of normality were determined using Q-Q plots and Shapiro tests, and where appropriate non-parametric tests were conducted. To test for significant differences between ancestral phenotypes and those isolates from the evolved populations Holm adjusted Wilcox tests were conducted. To test for the interacting effects of the community treatment and the presence of mucin upon the measure phenotypes linear mixed-effects models with mucin and co-occurring species identity and their interaction as main effects and replicate population as a random effect to account for repeated measures were conducted using the *{lme4}* package in R. For multivariant analysis of evolved phenotypes principal component analysis was conducted on the data following standardisation. Kaiser’s criterion and Cattell–Nelson–Gorsuch CNG Indices indicated that the first three components should be retained. In order to confirm separation of treatments was not masked by orthogonal signals in the single PCA presented in Fig. 1, individual PCAs were conducted for each treatment group separately which resulted in similar clustering, indicating that mucin had no effect on the multivariate phenotype in the absence of co-occurring species and in the three species communities (SM + SA), but altered the phenotypes in each of the two species communities (SM or SA). Pairwise correlation between phenotypic traits was calculated using Kendall Rank Correlation Coefficient. Within treatment diversity was calculated using multivariant homogeneity of group dispersions, which provides a multivariate analogue of Levene’s test for homogeneity of variances [62, 63]. Euclidian distances between each isolates’ phenotypes were reduced to principal coordinates and the Euclidian distance of each isolate from its treatment’s centroid was calculated to determine within treatment diversity. To analyse the diversification of individual phenotypic traits the median absolute deviation was calculated for each of the eight treatment groups. To test for significant differences in diversification between treatment groups in each trait Brown–Forsythe test were conducted.

To test if the mutations observed within each treatment had significantly different variances, a multivariate homogeneity of groups variances test was conducted [62]. A Euclidean distance matrix was constructed based on the binary presence and absence of mutation at each mutated loci across all treatment populations [64]. Homogeneity of variances between treatments was then calculated using “betadisper” in the R package *{vegan}*. To determine if different evolutionary treatments resulted in different sets of mutations a permutational multivariate analysis of variance was conducted using “adonis2” in the *{vegan}* package. The data were then partitioned into sets to test for significant differences between treatment groups and multiple testing was corrected for using Bonferroni correction. To test if the observed dS/dN ratio was significantly different from expected Chi-squared and Fisher’s Exact tests were conducted.

## Results

### *P. aeruginosa* competitively excluded *S. maltophilia* and *S. aureus* during each growth cycle

We first explored how the presence and identity of co-occurring species affected *P. aeruginosa* population densities, which could be indicative of competitive, neutral, or facilitative interactions. A mixed effects linear model with the presence of mucin, co-occurring species and transfer as fixed effects showed that the change in population density of *P. aeruginosa* was dependent upon both the presence of mucin and the presence of co-occurring species (Fig. S2, Co-occurring:Mucin:Transfer F_3_ = 3.0108, *P* < 0.05). However, only the presence of *S. maltophilia* in the absence of mucin resulted in a significantly reduction in *P. aeruginosa* density through time relative the *P. aeruginosa* alone treatment (Multiple comparison with Tukey adjusted *P*-values, −mucin:PA, −mucin:SM *P* < 0.001). In contrast, the densities of both *S. maltophilia* and *S. aureus* were significantly reduced when cultured with *P. aeruginosa* (Fig. S3), resulting in 95% reduction with *S. aureus* (mean SA density pre-transfer 1.61 × 10^7^ ± 3.09 × 10^6^ CFU/ml) and 10.5% reduction in case of *S. maltophilia* (mean SM density pre-transfer 1.09 × 10^8^ ± 1.54 × 10^7^ CFU/ml). Together these results suggest that while only *S. maltophilia* had slightly negative effect on *P. aeruginosa* densities in the absence of mucin, the presence of *P. aeruginosa* significantly reduced the densities of *S. aureus* and *S. maltophilia*, indicative of potential asymmetric competition. Thus, the competition was mainly unidirectional, where *P. aeruginosa* competitively excluded both *S. aureus* and *S. maltophilia* at each growth cycle.

### The presence of co-occurring species and mucin together interact in changing *P. aeruginosa* phenotypic evolutionary trajectory

To determine the evolutionary impact of the presence of co-occurring species upon *P. aeruginosa,* 12 evolved *P. aeruginosa* isolates were randomly sampled from each of the 48 evolved populations (8 treatments, 6 independent replicates each) upon completion of the transfer experiment (130 generations) and screened for changes in pathoadaptive traits including pyoverdine, pyocyanin, elastase, and protease production, adherence, growth dynamics, antibiotic sensitivity and metabolism (Figs S4 and S5). While there was no significant change in antibiotic sensitivity to five commonly used anti-pseudomonal drugs (Fig. S6), or major changes in ability to utilize the carbon and nitrogen sources present in SCFM (Figs S7 and S8), we observed significant changes in secreted products, adherence and growth rates in evolved *P. aeruginosa* isolates (Fig. 1 A-D, Fig. S4, Holm adjusted Wilcox test Table S1). These phenotypic adaptations were highly similar across independently evolving replicate populations within most treatments (Fig. 1E, Fig. S5), indicating a robust and parallel response in trait adaptation to the presence of co-occurring species. However, divergence in virulence factors (elastase, pyoverdine and adherence) was observed among populations evolving in the presence of *S. aureus* or *S. aureus* plus *S. maltophilia* in the presence of mucin (Fig. S5).

The production of virulence factors and growth dynamics of *P. aeruginosa* isolates evolved along different trajectories, depending on the presence of co-occurring species and mucin (Fig. 1, linear mixed-effects model, Table S2). In the absence of co-occurring species, all evolved *P. aeruginosa* isolates moved in a similar direction in the phenotypic trait space, while in the presence of co-occurring species, isolates showed distinct trajectories and occupied a wider range of different phenotypes relative to the *P. aeruginosa* evolving alone control treatment (Fig. 1). For example, while pyoverdine production increased, and elastase production decreased, in the absence of co-occurring species (Fig. 1A non-filled points), the presence of SA or SM resulted in an increase in elastase production but reduced pyocyanin production and reduced adherence and growth rates (Fig. 1B and C non-filled points; changes relative to the common ancestor). A similar trend was also observed in the three species community (Fig. 1D non-filled points), with *P. aeruginosa* isolates displaying increased elastase production, which was coupled with decreased adherence. Consequently, there was a significant positive correlation between adherence and pyoverdine production, together with a negative correlation between elastase production and adherence, and pyoverdine and elastase production across all treatments (Fig. 1F). Thus, the presence of *S. aureus* and *S. maltophilia* appears to maintain a wider range of pathoadaptive *P. aeruginosa* phenotypes at the population level compared to isolates evolving in the absence of these species.

The presence of mucin alone had little effect on the evolved *P. aeruginosa* phenotypes (Fig. 1A, compare unfilled and filled points). However, we found significant interactions where the presence of mucin changed the trajectory of phenotypic trait evolution within the pairwise treatments (Fig. 1B and C, compare unfilled and filled points; Linear Mixed-Effects Model, Table S2). For example, the presence of mucin in the *S. aureus* and *S. maltophilia* pairwise communities lead to phenotypes that resembled those of *P. aeruginosa* isolates evolving in the absence of these species (Fig. 1A-C filled points). This was primarily due to reduction in elastase production, which was coupled with increases in adherence and pyoverdine production. In contrast, mucin had lesser effect on the evolved phenotypes in the three-species communities (SM + SA, Fig. 1D, compare filled and unfilled points), with *P. aeruginosa* isolates maintaining high protease and elastase production, decreased adherence, pyoverdine and pyocyanin production in the presence of mucin. The presence of mucin hence appeared to constrain the selection imposed by the co-occurring species, whereas the evolved phenotypes were less affected by the mucin when both *S. aureus* and *S. maltophilia* were present.

### The presence of co-occurring species and mucin increase within-treatment diversification

**Figure 2:**
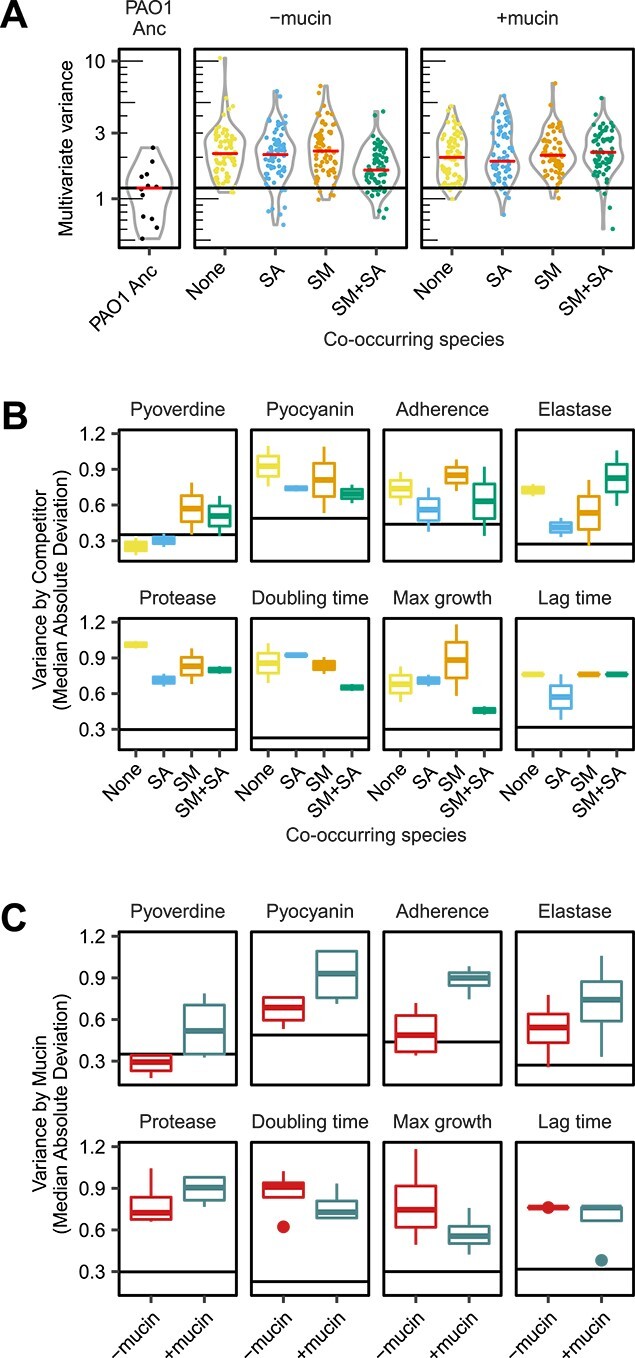
Phenotypic diversity increases across all treatments. Multivariant homogeneity of group diversity in phenotype within treatments. (A) Points show Euclidian distance to treatment centroids for each isolate (between population diversity). Ancestral strain multivariant diversity is displayed in the left facet, the horizonal line shows the median distance to centroid for the ancestor. The violin overlays show the distribution of diversity within treatments and horizontal lines within the violin plots show treatment median distance to centroids. Points coloured by co-occurring species identity, no co-occurring species (none), *S. maltophilia* (SM), *S. aureus* (SA), *S. maltophilia* plus *S. aureus* (SM + SA). (B) Boxplots showing the variance (absolute deviation) in phenotypic traits within community treatment groups, (C) Boxplots showing the variance (absolute deviation) in phenotypes within mucin environments. In all boxplots, the midpoint shows the median absolute deviation (MAD) score of each phenotypic trait, the lower and upper hinges correspond to the first and third quartiles and the upper and lower whiskers extend to 1.5× interquartile range of the deviation within treatment groups. The horizontal line shows the median absolute deviation in the ancestral strain trait measurements.

We next assessed if the presence of co-occurring species or mucin influenced the diversification of evolved *P. aeruginosa* phenotypes within treatments. First, we calculated within treatment diversity using multivariant homogeneity of group dispersions, providing a multivariate analogue of Levene’s test for homogeneity of variances. Euclidian distances between each isolates’ phenotypes were reduced to principal coordinates (Fig. S10) and the Euclidian distance of each isolate from its treatment’s centroid was calculated to determine within treatment diversity. We found that within-treatment *P. aeruginosa* diversity increased equally across all treatments compared to the ancestral strain, except for SM + SA treatment in the absence of mucin, where a smaller increase was observed (Fig. 2A, Multivariate homogeneity of groups variance, permutation test F_8,575_ = 4.91 *P* < 0.01, Tukey post-hock tests, SM + SA-SCFM vs ancestor, *P* = 0.666, all other treatments *P* < 0.05). Overall, the diversification of individual traits relative to the ancestral strain was clear with protease production and growth rate, which increased significantly across all treatments (pairwise Wilcoxon rank test, PAO1 Anc vs Evolved, *P* < 0.05, Holm adjusted). Moreover, the co-occurring species identity had significant effects on the diversification of pyoverdine and pyocyanin production, as well as on adherence and growth rates (Fig. 2B, Brown–Forsythe tests Table S3). The presence of mucin resulted in increased diversification regarding pyoverdine, pyocyanin and elastase production and adherence overall (Fig. 2C, Brown–Forsythe tests Table S3). Interestingly, *P. aeruginosa* strains evolved in the presence of co-occurring species displayed lower diversification regarding pyocyanin and protease production relative to strains evolved in their absence, indicative of strong parallel evolution within these treatments. Together, these results suggest that both the presence of mucin and co-occurring species affected the diversification of virulence associated traits of *P. aeruginosa*.

### Phenotypic and molecular adaptation show convergent evolutionary trajectories

To examine the genetic basis of phenotypic diversification, we conducted whole population genome sequencing for each evolved *P. aeruginosa* replicate population. In total, we observed 489 mutations across 158 unique loci. Across all observed mutations, 39.1% were non-synonymous, 36.4% intergenic, 12.3% synonymous, 5.1% deletions (non-frameshift), 2.6% gain of stop codons, 2.5% frame-shift inducing insertions or duplications, 1.6% frame-shift inducing deletions, and 0.4% duplications (non-frameshift) (Fig. S11, Supplementary Table S4). Most mutations within protein coding genes occurred at low frequency, with 84.2% of mutations observed at a frequency below 20% (detection threshold 5% frequency, Fig. S12). These variants had the expected number of synonymous mutations (χ^2^ = 0.48, *P* = 0.49, 22.1%, 58 out of 262) given that 25.2% of all possible variants within the coding genes of PAO1 are synonymous [65]. In contrast, all 49 mutations across 23 unique loci occurring at a frequency >20% (Fig. S12) were significantly overrepresented by non-synonymous mutations (Fisher’s Exact test odds ratio = 0.134, *P* = 0.007, 47 out of 49), indicative of positive selection at these loci. Genetic diversity, measured as the mean within population heterozygosity [48], was not significantly different between treatments (ANOVA, mucin F_1_ = 0.274, *P =* 0.603, Co-occurring species F_3_ = 0.04, *P* = 0.989, mucin × co-occurring species F_3_ = 1.68, *P* = 0.182, Fig. S13), consistent with the observed phenotypic diversification across all treatments. Additionally, the average heterozygosity was >0 across all treatments (*t*-test, *P* < 0.01 for all treatments), suggesting that mutational supply was high across all conditions. Together, these analyses suggest that most mutations were under weak selection, had not had sufficient time to reach fixation, or arose via genetic drift or hitchhiked along with other large-effect mutations associated with adaptive divergence.

**Figure 3:**
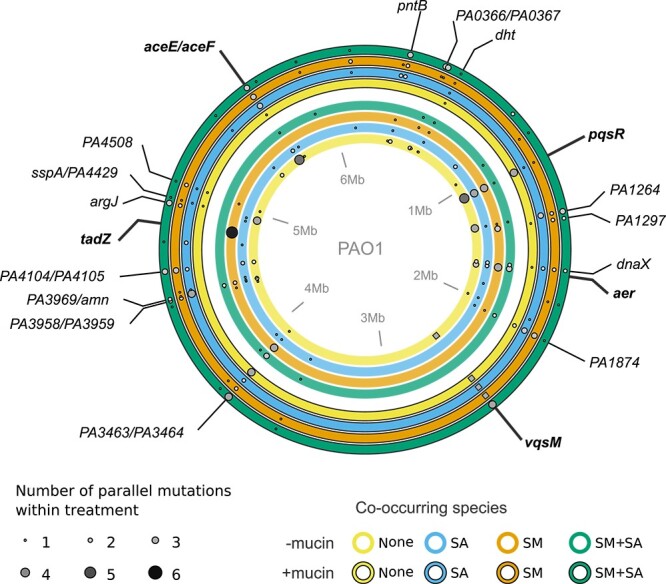
Genomic mutations show co-occurring species-specific profiles in the absence of mucin. Parallel mutations observed in evolved populations across treatments. Concentric circles represent *P. aeruginosa* genomes from each treatment, points show parallel mutations that occur three or more times across treatment replicates in whole population sequencing of communities at the end of the experiment; only variants that occur in at least six replicate populations are labelled. Inner rings represent treatments with no mucin, outlined outer rings represent treatments with mucin. Circle colours denote the co-occurring species that were present during the selection experiment (yellow none, blue *S. aureus (SA)*, orange *S. maltophilia (SM)* and green *S. maltophilia* plus *S. aureus (SM + SA)*). Point sizes and shade show the number of independent replicate populations in which the mutations were observed within treatment. Mutations labelled in bold are treatment-specific, squares represent deletions in the PA2228-*vqsM*-*qsrO* operon. For a full list of mutations see Supplementary Table S4.

Next, we determined if different sets of mutations evolved between treatments, indicative of independent evolutionary trajectories. While neither the presence of co-occurring species, or the presence of mucin, had a significant effect on the number of mutations (ANOVA, treatment *F*_3,40_ = 0.201, *P* = 0.89, mucin *F*_1,40_ = 0.004, *P* = 0.95, treatment × mucin *F*_3,40_ = 1.6, *P* = 0.197), mutations occurred in different loci between treatments (Fig. 3, permutational ANOVA, permutation test: *F*_7,40_ = 1.3553, *P* < 0.01, Bonferroni corrected, 1000 permutations). Populations from the pairwise (SA or SM) and three species (SM + SA) treatments evolving in the absence of mucin had significantly different sets of mutations from each other (permutational ANOVA, permutation test: *F*_3,20_ = 1.962, *P* < 0.05, Bonferroni corrected, 1000 permutations). In contrast, evolution in the presence of mucin resulted in non-significant differences in the loci between treatments (permutational ANOVA, permutation test: *F*_1,22_ = 1.368, *P* = 0.24, Bonferroni corrected). Within treatments, the presence of mucin only altered the mutated loci in the SM treatment (permutational ANOVA, permutation test: *F*_1,11_ = 1.891, *P* < 0.05, Bonferroni corrected), while the mutated loci within all other treatments were not affected by the presence of mucin. Together, these findings suggest that unique sets of mutations were selected in different communities irrespective of the presence of mucin, except for the SM treatment.

### Community-specific parallel evolution occurs in virulence-associated genes

Next, we explored the treatment-specific nonsynonymous mutations. Overall, the loci mutated with the highest frequency were predominately associated with genes involved with quorum sensing and virulence factor production (*pqsR, vqsM*), adherence/biofilm production and motility (*tadZ*, *pilB, pilQ pilY, pilZ, aer*), metabolism (*aceE/aceF*), and DNA replication (*dnaX*) (Fig. 3). Mutations at loci present across all treatments, such as missense mutations in *dnaX*, PA1874, encoding a hypothetical ABC transporter, and several intergenic mutations, suggest convergent evolution due to shared experimental lab conditions (e.g., SCFM medium [48]). In contrast, other mutations occurred exclusively in the absence of mucin independent of co-occurring species identity, which included the loss of function and missense mutations within the aerotaxis receptor *aer* (Fig. 3). However, the presence of the most frequently mutated genes was specific to both the community composition and the presence of mucin. For example, highly parallel non-synonymous mutations in *tadZ,* which encodes tight adherence pili, were only present in SM treatments in the absence of mucin. Likewise, non-synonymous loss of function mutations in other adherence and motility associated pilus genes (*pilB, pilS pilY, pilQ*, and *pilZ*) were only detected in the absence of co-occurring species and mucin and associated with small colony variant morphology. Intergenic mutations between pyruvate dehydrogenase (*aceE*) and dihydrolipoamide acetyltransferase (*aceF*) also predominantly occurred when *P. aeruginosa* evolved alone in the absence of mucin (5/6 populations), indicating divergence in the targets of selection between treatments.

**Figure 4:**
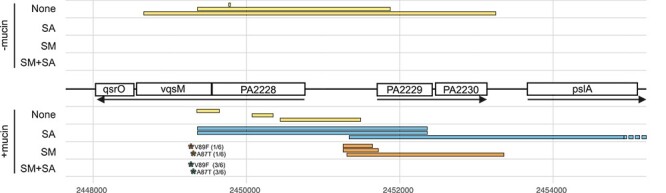
The presence of parallel deletions within the *PA2228-vqsM-qsrO* operon mirror separate phenotypic trajectories. Coloured lines represent treatment-specific deletions in three representative isolates from each treatment. Asterisks represent point mutations with numbers in brackets displaying the number of replicate populations in which the mutations occurred. Arrows show operon structure, lines coloured by treatment, no co-occurring species (none), *S. maltophilia* (SM), *S. aureus* (SA), *S. maltophilia* plus *S. aureus* (SM + SA). The deletion in isolate three in *S. aureus* (SA) + mucin treatment encompassed an additional 14 genes of the psl operon.

Missense mutations within *pqsR*, the transcriptional regulator of the *Pseudomonas* Quinolone Signal (PQS) system involved in the regulation of virulence factor production, biofilm formation and motility phenotypes [66], showed strong locus-level parallelism in the absence of mucin, occurring in the PA alone (5/6 populations), SA (4/6), SM (4/6), and SM + SA (1/6) treatments. However, mutations at this locus were underrepresented in the presence of mucin, particularly in the presence of *S. maltophilia* (mutations present in 1/6 populations) or *S. aureus* (mutations present in 1/6 populations) and never occurred in the presence of both species. Thus, the presence of mutations in *pqsR* appears to be predominately selected in the absence of mucin, particularly in the presence of the co-occurring species. Furthermore, the presence and type of mutations in the PA2228-*vqsM*-*qsrO* operon, a global regulator of quorum sensing mediated virulence factor production [67, 68], depended on the presence of co-occurring species and mucin (Fig. 4). Parallel deletions within PA2228 and *vqsM*, as well as the adjacent intergenic region, ranging from 22 bp frameshift inducing to whole gene loss (4593 bp) deletions, were observed only when *P. aeruginosa* evolved in the absence of co-occurring species in the absence of mucin (Fig. 4). Moreover, these deletions occurred also in all pairwise co-culture treatments in the presence of mucin, while highly parallel non-synonymous point mutations within *vqsM* gene were observed in the three-species communities in the presence of mucin. These results suggest that co-occurring species could maintain *P. aeruginosa* virulence in CF infections in the absence of mucin and when competing only with one species.

### Linking parallel mutations with observed phenotypic changes

**Figure 5:**
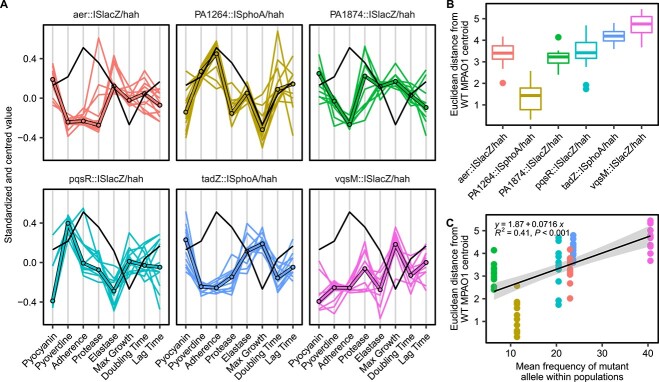
Transposon mutagenesis of most frequently mutated genes has the largest effect on phenotypes. (A) Parallel coordinate plot of standardized phenotypic measurements of transposon mutants within genes most frequently mutated across parallel populations. Bold line shows the median phenotype across independent biological replicates (n = 12), the coloured lines show each individual biological replicate, and the black line shows the median phenotype of the parental strain MPAO1 (n = 12). For non-standardized data see Fig. S14. (B) The Euclidian distance of transposon mutants’ phenotypes from their parental MPOA1 strain calculated from principal coordinates based on all measure phenotypic traits. Lower distance from wild-type MPAO1 indicates that the loss of function of the gene has a lower overall effect on the traits measured. (C) Correlation between the Euclidian distance of transposon mutants’ phenotypes from their parental MPOA1 and the mean frequency at which mutations arose within evolved populations, following the colour scheme of a and b.

To link parallel genetic changes with observed phenotypic variation, we phenotyped mutants with transposon insertions in genes that were observed to evolve in parallel across multiple independent replicate lines within treatments. Transposon mediated loss-of-function of *aer*, *pqsR*, *tadZ*, *vqsM*, PA1264, or PA1874 significantly affected at least one of the phenotypic traits measured (Fig. 5A and B, Fig. S14), with transposon mutagenesis predominately affecting virulence factor production. Importantly, there was a significant positive correlation between the strength of the effect of the mutation upon the resulting phenotypes (Euclidean distance of the multivariate phenotype from that of the ancestor) and the mean frequency at which the mutation occurred within evolved populations (Fig. 5C, R^2^ = 0.4, F_1,70_ = 48.18, *P* < 0.001). Loss of function transposon mutants within the quorum sensing regulators *vqsM* and *pqsR,* and the adherence factor *tadZ*, had the largest overall effect on phenotypes (Fig. 5B) and were also the most frequently mutated genes during the selection experiment (Fig. 5C). These results show that parallel mutations in genes with the largest effect on phenotypes were under strong selection, and that specifically, mutations within *pqsR* and the PA2228-*vqsM*-*qsrO* operon were the predominant drivers of the phenotypic trait evolution. The loss of function of *vqsM* in the transposon mutants resulted in decreased adherence, pyocyanin, pyoverdine, protease and elastase production (Fig. 5A). However, other than *vqsM*, the mutations that occurred during the selection experiment were not loss of function mutations hence their effect is difficult to predict. Parallel mutations within PA1264 were all synonymous variants and thus were likely of low effect, no predicted protein domains were present within *tadZ* or PA1874, and missense variants within *aer* occurred outside of predicted domains. In contrast, missense variants within *pqsR* occurred within the ligand-binding domain and therfore may interfere with its ability to bind PQS and HHQ which results in a confirmational change required for the activation of the *pqs* operon.

**Figure 6:**
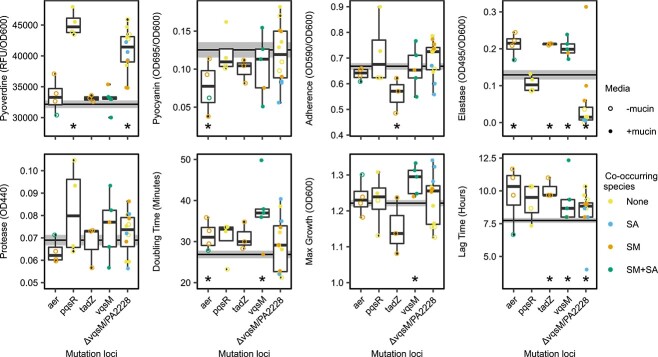
Parallel mutations led to similar phenotypic changes to those observed in the selection experiment. Phenotypes of individual evolved isolates with point mutations in the most commonly loci mutated during the evolution experiment. Points are coloured by the treatment from which they were isolated. Isolates were individually whole genome sequenced and selected based on containing a single mutation within the loci represented on the x-axis. Isolates labelled Δ*vqsM/*PA2228 had deletions ranging between 22 bp and 4593 bp within the quorum-sensing PA2228-*vqsM*-*qsrO* regulatory operon, while isolates labelled with *vqsM* had non-synonymous mutations within *vqsM* (A87T or V89F). The midpoint of box plots shows the median trait value, the lower and upper hinges correspond to the first and third quartiles and the upper and lower whiskers extend to 1.5× interquartile range. The horizontal black line shows the mean trait value in the ancestral strain, with the shaded area showing the standard error of the mean. *aer* N = 4, *pqsR* N = 4, *tadZ* N = 3, Δ*vqsM*/PA2228 operon N = 12, mutations outline in table S5. Stars show significance difference from the ancestral value (Wilcox test, *P* < 0.05, corrected for multiple testing using Holm–Bonferroni method).

As transposon mutagenesis does not replicate the type of mutations observed during the selection experiment, we also sequenced a single isolate from each of the evolved replicate populations to directly link genotypes to phenotypes (N = 6 per treatment). Most of the sequenced evolved isolates contained only a single mutation at one of the parallelly evolved treatment specific loci, which allowed us to associate evolved phenotypes with specific mutations (Table S5, Fig. 6). Non-synonymous mutations in *aer*, specific to populations evolving in the absence of mucin, significantly reduced pyocyanin production and increased elastase production similar to the *aer* transposon mutants (Fig. 5). In contrast, point mutations in *tadZ* in evolved isolates resulted in different phenotypes to the transposon mutants, only reducing adherence and increasing elastase production. Likewise, point mutations in *pqsR* in evolved isolates were different to those of the transposon mutants, resulting only increase in pyoverdine production. Deletions within the PA2228-*vqsM*-*qsrO* operon were specific to the populations which evolved phenotypes resembling those of *P. aeruginosa* evolving in the absence of co-occurring species (SM and SA in the presence of mucin) but absent in populations that displayed alternative phenotypic trajectories (SM, SA and SM + SA in the absence of mucin, and SM + SA in the presence of mucin). Indeed, deletions at this locus increased pyoverdine production and decreased elastase production and are likely a key driver in the different parallel evolutionary trajectories. Moreover, deletions within this locus contribute to the diversification in pyocyanin production and growth rates observed within these treatments (Fig. 6). Importantly, point mutations within *vqsM,* present within the populations evolving in the presence of SM + SA + mucin treatment resulted in a divergent phenotype with no effect on pyoverdine production, but significant increase in elastase production (Fig. 6). The observed parallel mutations hence led to similar phenotypic changes to those observed during the selection experiment, validating their role in driving the different evolutionary trajectories between treatments.

## Discussion

The evolution and diversification of *P. aeruginosa* within the lungs of pwCF enables lung-naïve, non-adapted strains to optimize their pathoadaptive fitness to the CF airways, leading to the long-term chronic infection of the lung and irreversible tissue damage [4]. This evolutionary diversification of *P. aeruginosa* takes place within an ecologically complex environment [3, 69], both in terms of species composition and physical environment. Here, we investigated the impact of community composition and the presence of mucin upon the *in vitro* evolution of lung-naïve *P. aeruginosa* within lung-like conditions. Our results show that the local microbial community can shape *P. aeruginosa* diversification and evolutionary trajectory, leading to differences in phenotypes between different experimental communities (Fig. 1A-E). The presence of mucin also affected this process, particularly within two species communities (Fig. 1B and C). In contrast, selection by communities consisting of both *S. aureus* and *S. maltophilia* were less affected by the addition of mucin (Fig. 1D), suggesting that the strength of biotic interactions is dependent on the growth of the environment. Loss of function mutations in major global virulence factor regulators was only present in the absence of other co-occurring species in the absence of mucin, or in two-species communities in the presence of mucin. This suggests that the loss of virulence often associated with the transition from acute to chronic infection may reflect a lack of selection by co-occurring microflora or sputum during the early stages of colonization.

How did the presence of mucin change the evolutionary trajectories of *P. aeruginosa* in the presence of co-occurring species? Mucins are high molecular weight glycoproteins that form a protective layer throughout the airways [43]; their dysregulation in CF airways contributes to the high viscosity and spatial heterogeneity of CF sputum [44]. The presence of mucin has previously been shown to drive phenotypic diversification of *P. aeruginosa* through reduced dispersal leading to divergent selection, local adaptation, and reduced evolutionary parallelism [13, 48]. In support of this, we also observed that the presence of mucin increased diversity, particularly in virulence related phenotypes (pyoverdine, pyocyanin, elastase production, and adherence). As mucin is not obviously utilized directly by *P. aeruginosa* as a nutrient source [70], the divergence between replicate populations in the presence of mucin was more likely due to changes in the intensity of local interactions, which could have resulted in greater within and between population diversity by creating spatial separation between species [71]. For example, polymicrobial chronic wound models of infection consisting of *P. aeruginosa* and *S. aureus* have been shown to allow the coexistence of the two species at high densities due to a patchy, segregated distribution of the two species [72]. The presence of mucin altered the *P. aeruginosa* evolutionary trajectory in pairwise cocultures. However, mucin had little effect on the evolutionary trajectory of *P. aeruginosa* in the presence of both *S. maltophilia* and *S. aureus*. This could be explained by relatively stronger interactions with *P. aeruginosa*, as both *S. maltophilia* and *S. aureus* reached higher densities in the three species compared to the pairwise community treatments (Fig. S3). Moreover, the presence of *S. aureus* initially increased *P. aeruginosa* density in the absence of mucin suggesting a facilitative interaction. However, species population densities fluctuated considerably during the experiment and were not directly linked with the magnitude of evolutionary changes (Fig. S3). Co-occurring species could hence impose strong selection on each other even in the absence of clear population density effects. In the future, it would be important to also study potential evolution between all community members. In our experiment, over 99.9% of SM and SA cells were refreshed at each transfer with ancestral strains, reducing the likelihood of their evolution. While we tried to analyse changes in evolved *S. aureus* and *S. maltophilia* variants at the end of the experiment, the read depth was not sufficient due to low relative abundances of these species. This indirectly suggests that the few remaining *S. maltophilia* and *S. aureus* cells that had opportunity to evolve during the experiment unlikely attained considerable fitness increases.

Differential selection of quorum sensing regulated virulence factor production played a key role in the different phenotypic evolutionary trajectories observed between treatments. Strong selection against QS-virulence factor production through deletions within the PA2228-*vqsM*-*qsrO* operon and adjacent ORFs (PA2229 and PA2230) were observed in the absence of co-occurring species (Fig. 4). Deletions within these genes generated strains deficient in QS-dependent gene expression [68], and were not observed in the presence of co-occurring species in the absence of mucin. However, in the presence of mucin deletion of the PA2228-*vqsM*-*qsrO* operon and adjacent ORFs occurred in parallel in populations from the *P. aeruginosa* alone and pairwise communities (SM or SA), but not in the three species communities (SM + SA). In addition, the presence of mucin altered the frequency of non-synonymous mutations within *pqsR*, the transcription regulator of the *pqsABCDE* operon which controls intercellular QS-related signals 2-heptyl-4-hydroxyquinoline N-oxide (HQNO), 4-hydroxy-2-heptylquinoline (HHQ), and *Pseudomonas* quinolone signal (3,4-dihydroxy-2-heptylquinoline [PQS]) [73]. Mutations within *pqsR* were underrepresented in the presence of mucin and co-occurring species (Fig. 3). These results support the recent findings of Luján *et al*. [19], who demonstrated that *pqsR*-regulated QS system is retained in the presence of competing taxa and mucin, likely due to the fitness benefits of retaining the redox agent pyocyanin, an effective anti-competitor toxin. Mutations in QS transcriptional regulators *pqsR* and *vqsM* are commonly observed during *in vitro* evolution of *P. aeruginosa* [18–20, 48, 74, 75], but mutations in these genes are rarely observed in chronic adapted strains isolated from CF sputum [5, 76]. Our results suggest that mutations within these QS transcriptional regulators are dependent upon the interaction between the community composition and the physical environment, whereby the presence of other species coupled with the presence of mucin provide strong selection for the retention of these QS transcriptional regulators. The maintenance of a competitive phenotype is likely to be important for the ability of *P. aeruginosa* to coexist with co-residing pathogens. Interspecies competition may also help to explain how *P. aeruginosa* comes to dominate the microbiome of the CF lung despite long-term selective pressures selecting for the loss of virulence in chronic adapted isolates [77].

Treatment-specific phenotypic adaptation and diversification was associated with distinct molecular changes between treatments. These included several mutations at loci that have been identified to occur during the adaptation of *P. aeruginosa* within chronic infections, such as mutations in *aceE, aceF*, *tadZ*, pilus (*pil*) genes, and aerotaxis genes [9, 78, 79]. Mutations in *aceE* and *aceF* decouple glucose metabolism from the tricarboxylic acid (TCA) cycle, and are characterized as pathoadaptations [4], resulting in increased reliance on amino acid metabolism. Intergenic *aceE/aceF* mutations were overrepresented across all treatments in the presence of mucin (Fig. 3), but this was not coupled with a significant change catabolism within our experiments (Fig. S7). Likewise, we observed highly parallel mutations in the tight adherence gene *tadZ* in the presence of *S. maltophilia*. The *tad* genes are essential for biofilm formation, colonization and pathogenesis in *Pseudomonas* [80] with mutations previously being identified in *P. aeruginosa* strains responsible for prolonged hospital outbreaks [79]. Few mutations reached fixation during the evolution experiment; many more mutations occurred at low frequency or were not parallel between evolving populations, suggesting relatively weak selection, particularly in the presence of mucin. Periods of weak selection coupled with low mixing due to the physical environment will likely provide a basis of standing genetic and phenotypic diversity upon which stronger selection pressures (e.g. immune system, antibiotics) can act. In the CF lung such periods of weak selection, between antibiotic treatments for example, may be an important generator of diversity allowing for prolonged infection [81] and while *P. aeruginosa* often end up dominating the lung microbiomes of pwCF, the temporal dynamics of microbiomes are highly dynamic over shorter time scales [82]. Our results show that the evolutionary trajectory of *P. aeruginosa* is highly sensitive to the presence of other species and mucin, which could interactively determine *P. aeruginosa* potential for pathoadaptation in CF-lung infections.

Although we observed clear changes in virulence traits in our experimental system, we did not observe changes in other phenotypes commonly associated with chronic CF infection such as changes in susceptibility to antibiotics (Fig. S6), major changes in regulatory systems (*rpoN*, *gacS,* or *gacA*), significant changes in catabolism (Fig. S7, S8), mucoid phenotypes, or the evolution of hypermutators via mutations in miss-match repair genes. These results suggest that evolution in SCFM, media designed to mimic the nutritional environment of CF-sputum, or the presence of co-occurring species, do not impose selective pressures that influence the evolution of these traits. Rather, other selective pressures such as the intense use of antibiotics, and the highly fluctuating selective pressures that treatment regimens impose [83], the host immune system [84], hypoxic conditions [85], or competition from other species not included in our experimental system are likely to affect these phenotypic traits. The environment of the CF-lung is more complex and variable, both spatially and temporally, and such factors are not captured within our model. Further work on the impact of such factors upon the evolution of *P. aeruginosa* is required.

Our findings have important implications on understanding the role of microbial ecology in driving *P. aeruginosa* adaptation in CF-lung. Ecological patterning is associated with lung function in CF; decreased microbial diversity is associated with decreased lung function and dominance of the lung microbiome by CF pathogens [86]. The structure of the surrounding microbial community and presence of mucin determines the effect of interspecies interactions upon *P. aeruginosa*, which leads to divergent phenotypic and genetic diversification and adaptation. This suggests that the evolution of *P. aeruginosa* in the CF lung could be highly sensitive to the underlying ecological conditions, which may help to explain the observed within and between-patient variation in diversity [40]. What such patterns of interaction between the physical environment and co-occurring pathogens within other polymicrobial infections such as UTIs or wound infections, or with co-occurring non-pathogenic, hold remains to be seen. Although parallel pathoadaptation in chronic *P. aeruginosa* phenotypes is common, high virulence sub-populations are often maintained during chronic infection [41], and *P. aeruginosa* virulence factors can be detected in sputum samples throughout chronic infection [87]. Our findings suggest that the diversity of outcomes during adaptation in the CF lung may be driven by both the local microbial community composition and its interaction with the physical environment. Highly virulent sub-populations of *P. aeruginosa* may be maintained within-patients due to intense local interspecies interactions between CF pathogens, while at a macro-scale diversity could be limited by other strong selective pressures such as from the host immune system, resulting in the selection for the typical phenotypes associated with chronic infection. Ultimately, variation in the composition of the co-occurring bacterial species together with the mucosal environment, which may modulate the strength of interspecies interactions, can drive the diversification and adaptation of *P. aeruginosa* during lung infection.

## Conflicts of interest

The authors declared no conflict of interests.

## Funding

This work was supported and funded by The Wellcome Trust through the Centre for Future Health (CFH) at the University of York (204829/Z/16/A) and the Sir Henry Wellcome Postdoctoral Fellowship programme (221663/Z/20/Z).

## Data availability

Add data generated in this project is available on FigShare (10.6084/m9.figshare.24212064). All sequence data is available on EBI, accession PRJEB62433.

## Supplementary Material

SI_ycae043

Table_S1_ycae043

Table_S2_ycae043

Table_S3_ycae043

Table_S4_ycae043

Table_S5_ycae043

Table_S6_ycae043
