# Supplementary material for: Co-occurring microflora and mucin drive Pseudomonas aeruginosa diversification and pathoadaptation
Source: ISME Commun. 2024 Mar 28;4(1):ycae043. doi: 10.1093/ismeco/ycae043 (PMC11067959; doi:10.1093/ismeco/ycae043)
Supplement: SI_ycae043 [file si_ycae043.pdf]

Supplementary information for:

# **Community structure and local environment drive *Pseudomonas aeruginosa* diversification and pathoadaptation**

Michael J. Bottery, Helle Krogh Johansen, Jon W. Pitchford & Ville-Petri Friman

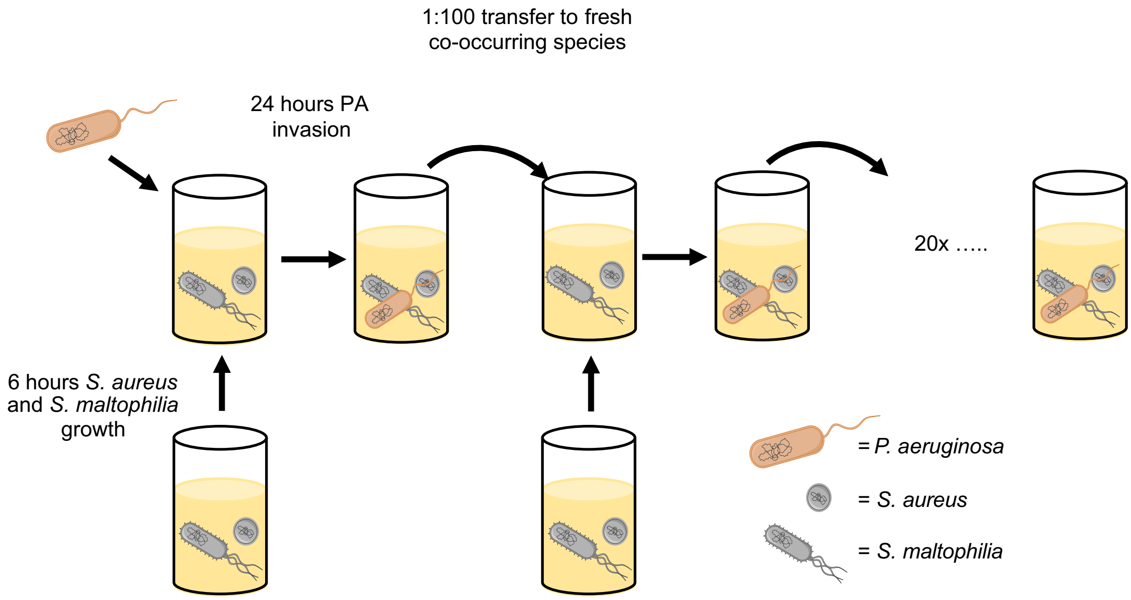


**Figure S1** We evolved *P. aeruginosa* in the presence or absence of commonly co-occurring species *Staphylococcus aureus* or *Stenotrophomonas maltophilia* or both species in Synthetic Cystic Fibrosis Media (SCFM), with or without the addition of mucin. The design of the experiment was fully factorial. Interacting species were pre-grown for 6 hours before the addition of the lung naive *P. aeruginosa* strain PAO1, which was allowed to invade the population and grow for 24 hours. At the end of the growth period the population was transferred one in one hundred into fresh media containing pre-grown fresh co-occurring species, allowing *P. aeruginosa* to invade once again. The populations were transferred a total of 20 times which resulted in approximately 135 *P. aeruginosa* generations. Without pre-growth, *S. aureus* and *S. maltophilia* would have been driven to extinction. Each of the 8 evolution treatments (Media: -mucin or +mucin, No co-occurring species [None], *S. maltophilia* [SM], *S. aureus* [SA], or *S. maltophilia* + *S. aureus* [SM+SA]) had 6 independent evolving lines, each inoculated with isogenic *P. aeruginosa*. The densities of *P. aeruginosa* were monitored daily throughout the experiment though plating onto Pseudomonas Selective

Agar, and the density of the co-occurring species were measured every 5 transfers through plating onto Mannitol Salt Agar or Stenotrophomonas selective media (Low Salt LB + 16 ug/ml imipenem and 5 ug/ml vancomycin) (Figure S3). Following evolution, 12 *P. aeruginosa* colonies were isolated from each evolved population and phenotypes were measured (pyoverdine, pyocyanin, adherence, elastase, protease, growth rate, max growth (OD), lag time [Figure 1], antibiotic MICs [tobramycin, meropenem, ciprofloxacin, aztreonam, colistin, Figure S5]). Whole population sequencing of the end point populations was conducted, and reads were mapped to a combined reference containing PAO1 reference genome, and *de novo* assembled genomes of *S. aureus* and *S. maltophilia* (Figure 3, Figure S10, S11).

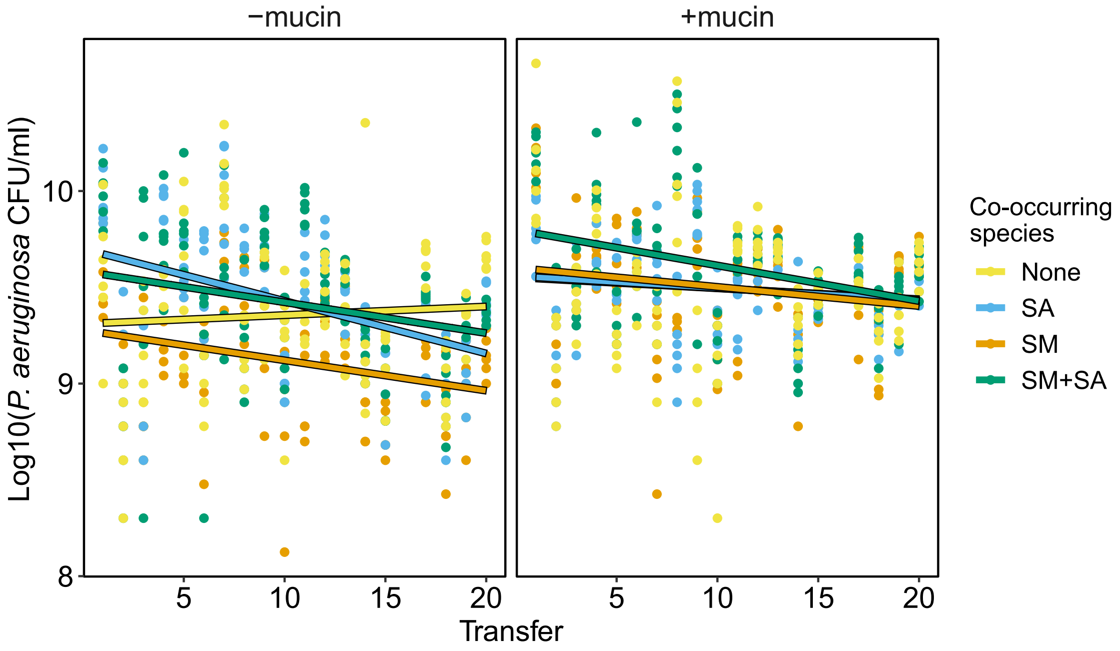


**Figure S2** Correlation between *P. aeruginosa* cell density [$\log_{10}$(CFU/ml)] and transfer. Lines show linear model fit coloured by co-occurring species identity, lines with black outline show significant correlation ($p < 0.05$). Each point shows the density of one of six independently evolving populations. Plot faceted by the addition of mucin.

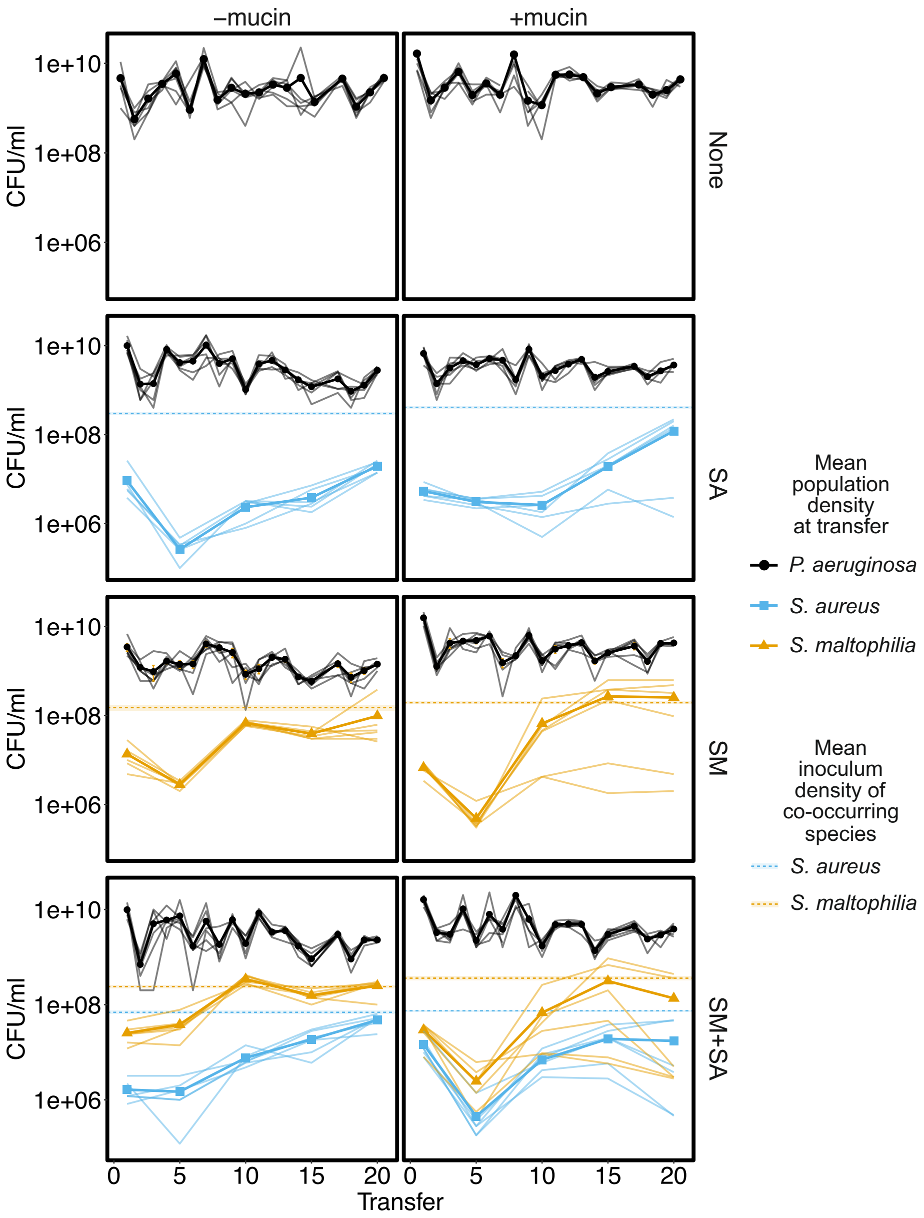


**Figure S3** Mean population density through time. Plots are faceted by the presence of mucin and co-occurring species identity. Black lines show the mean density of *P. aeruginosa*, the blue lines with square points shows the mean density of *S. aureus* and the orange lines with triangle points shows the mean density of *S. maltophilia* at the end of each 24 hour growth cycle before transfer into fresh media. Population dynamics in each of the 6 independent populations are shown as light lines. The horizonal dotted blue and orange lines in the SA, SM and SM+SA facets denote the mean density of *S. aureus* or *S. maltophilia* after the 6 hours pre-growth period when *P. aeruginosa* was transferred into the cultures (see methods).

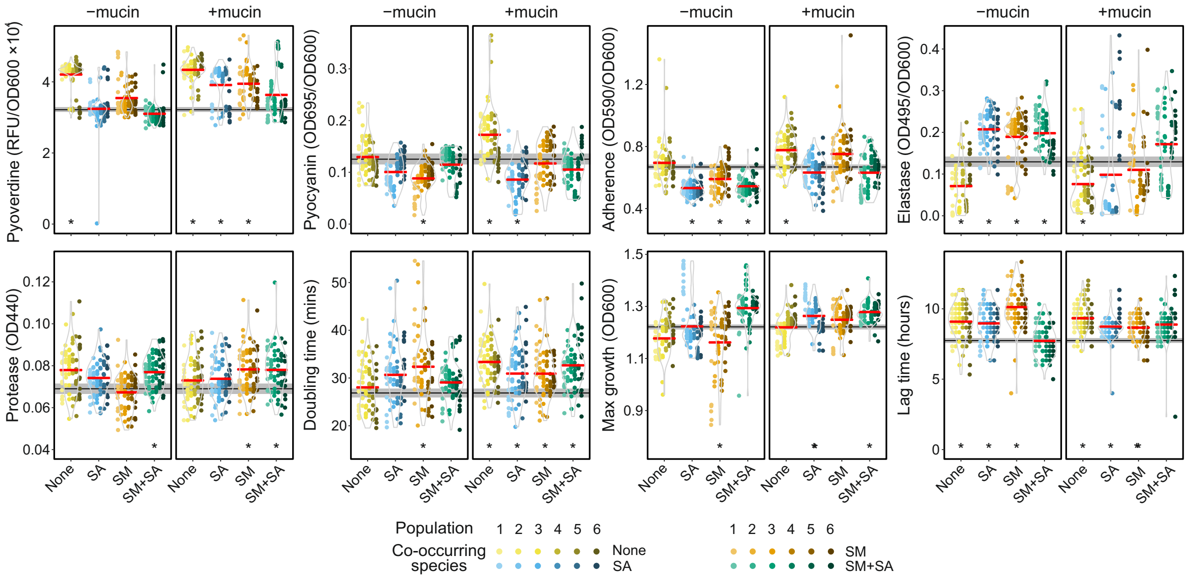


**Figure S4** The phenotypes of evolved *P. aeruginosa* isolates (n=12) within each population and condition. The phenotypes of twelve isolates from each of the six independent populations are shown as individual points, the hue indicates the population from which the colony was isolated from. The violin overlays show the distribution of the phenotypes within treatments and the red horizontal line shows the grand median across populations within treatments (n = 72). The horizontal black line shows the mean phenotype of the ancestral clone, the grey shaded areas show the standard error of the mean (n = 12). Stars indicate significant difference between the evolved phenotype and the ancestral phenotype (Wilcox Test, $p < 0.05$, corrected for multiple testing using Holm–Bonferroni method).

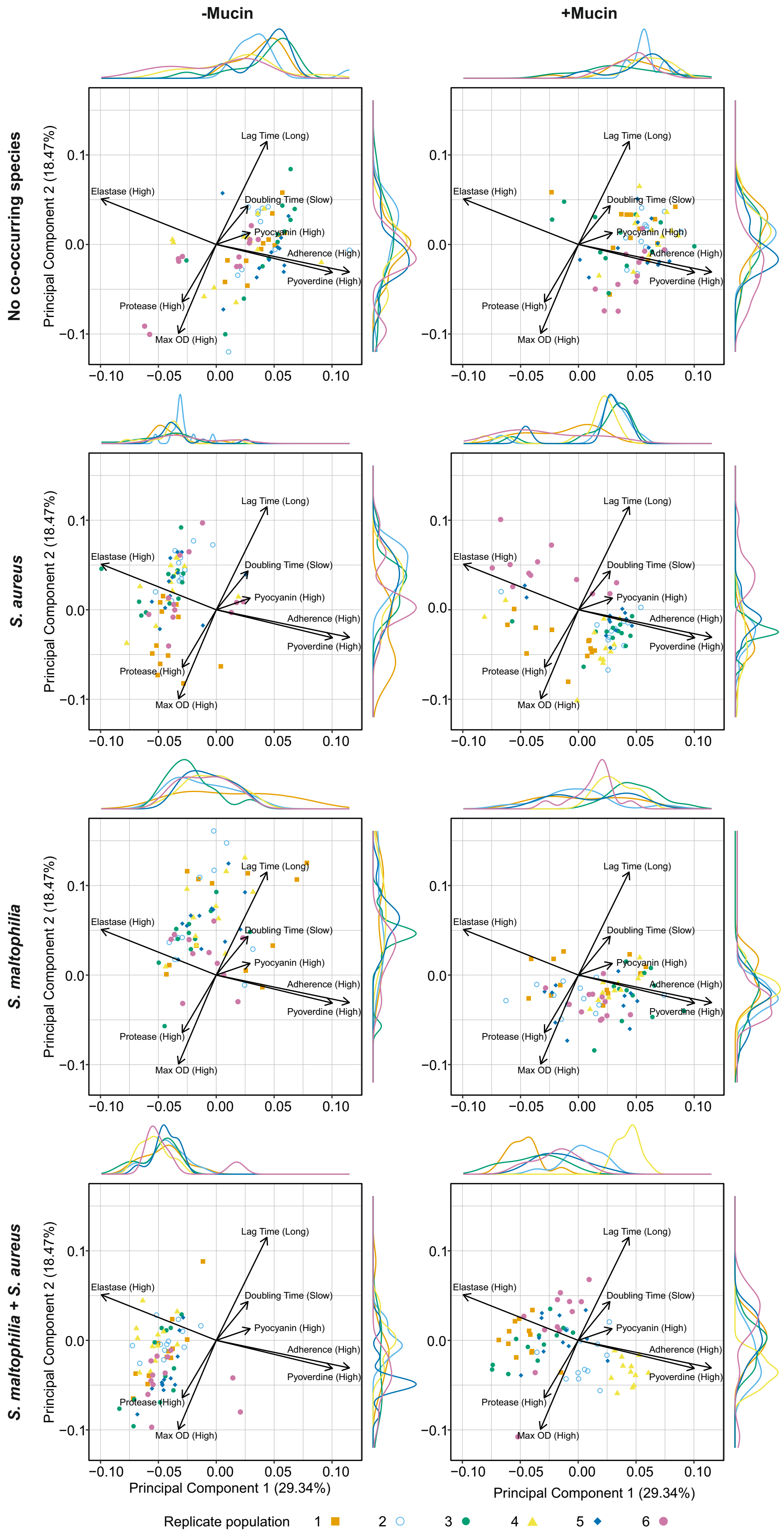

-Mucin
+Mucin
No co-occurring species
S. aureus
S. maltophilia
S. maltophilia + S. aureus
Principal Component 2 (18.47%)
Principal Component 1 (29.34%)
Lag Time (Long)
Elastase (High)
Doubling Time (Slow)
Pyocyanin (High)
Adherence (High)
Pyoverdine (High)
Protease (High)
Max OD (High)
Replicate population
1
2
3
4
5
6

**Figure S5** Ordination plots from a principal component analysis of phenotypic trait measurements of evolved isolates (n = 12) with each treatment coloured separately. Plots facetted by co-occurring species and mucin treatment. The first two components describe 48% of the variance. The overlayed loadings plot (grey arrows) shows the extent to which each phenotypic trait contributes to each principal component and the separation of the points. Points represent individual isolates, the density of points across each axis is shown at the top (PC1) and right (PC2) of the plots.

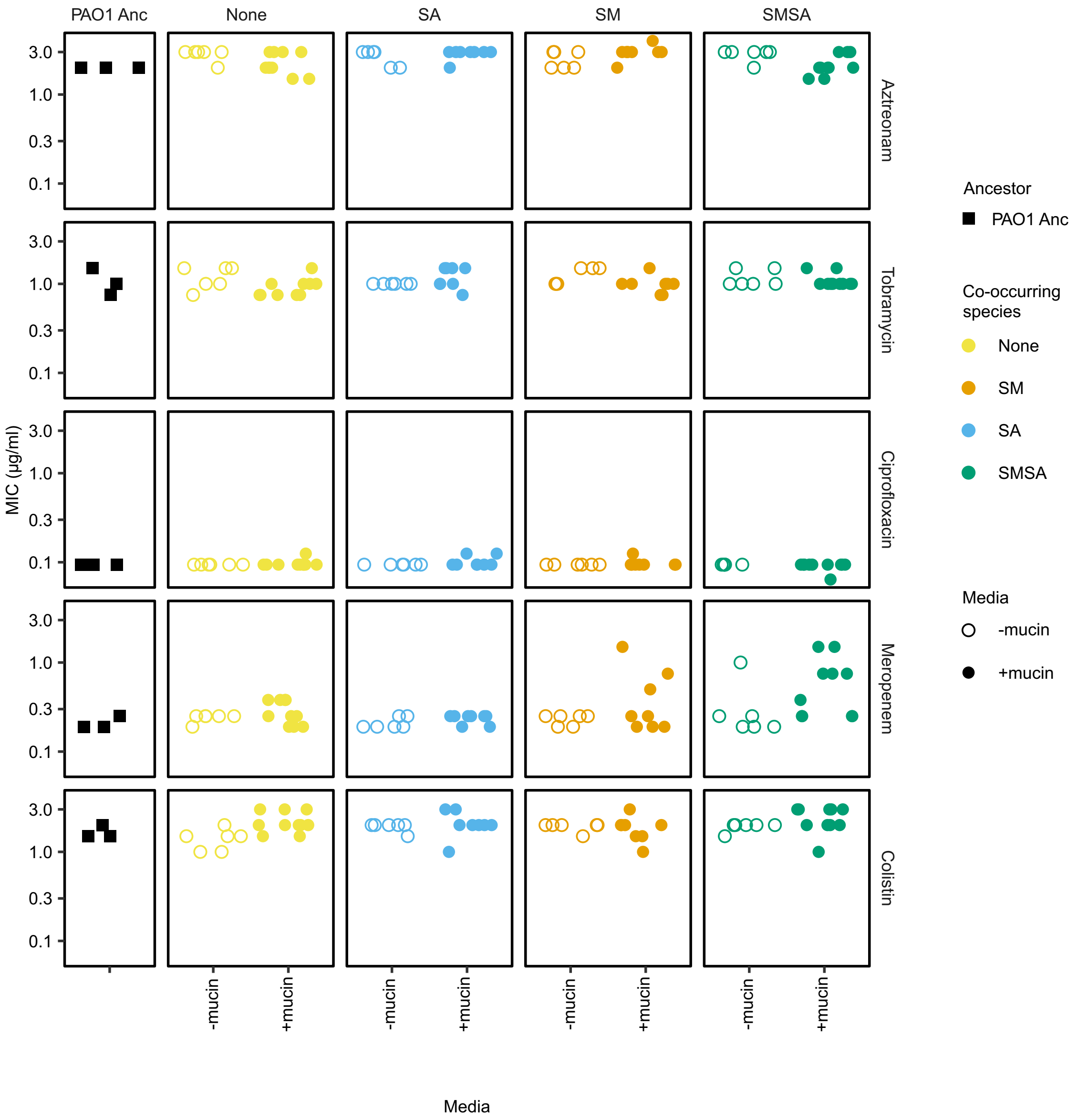


**Figure S6** MICs of at least 6 randomly selected evolved isolates to aztreonam, tobramycin, ciprofloxacin, meropenem and colistin. MICs measured using Etest strips. Black points show the MICs of the ancestral *P. aeruginosa* strain (N=3); coloured points show the MICs measured in the evolved isolates (minimum of N=6), isolates from -mucin treatments are represented with open points and from +mucin treatments with filled points.

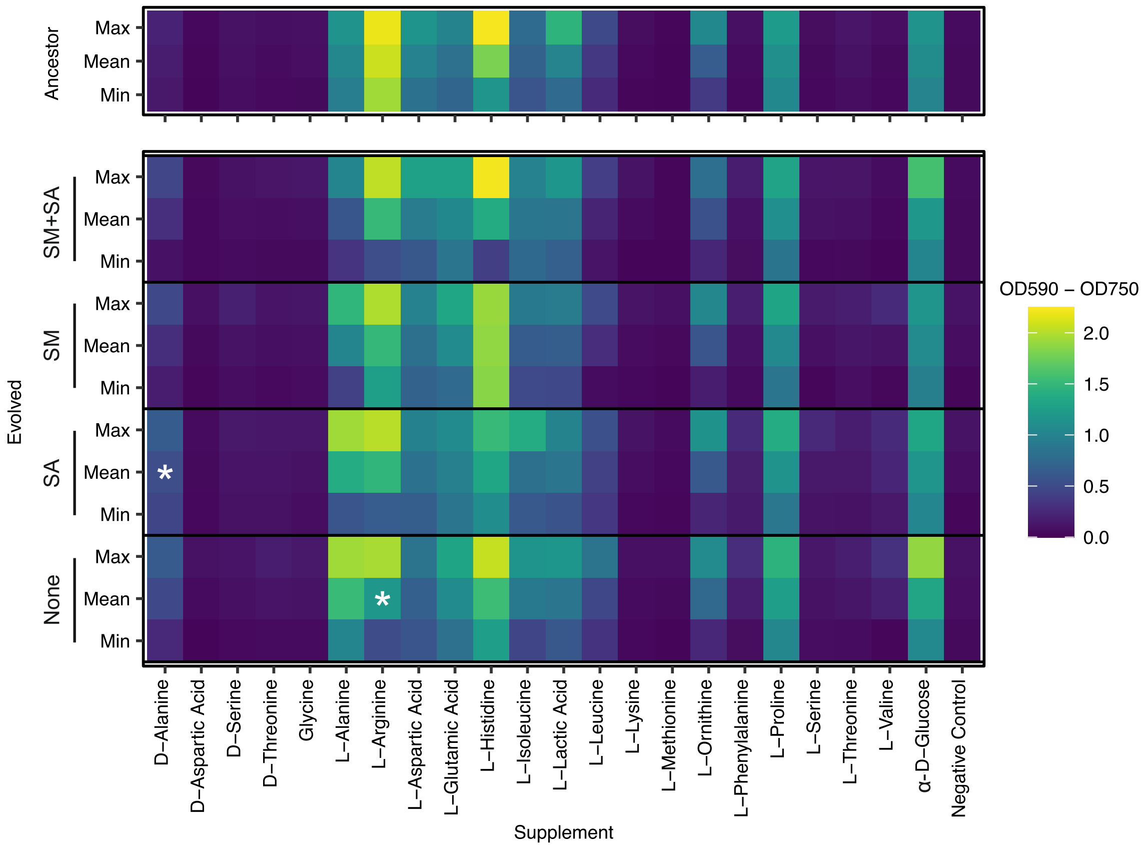


**Figure S7** Growth on sole carbon sources present in SCFM media. Mean, minimum and maximum growth of three replicates of ancestral *P. aeruginosa*, and mean, minimum and maximum growth of five colonies isolated from five different randomly selected populations evolved in the presence of mucin from each community. Stars show significant difference from ancestor (T-test, corrected for multiple testing using Holm–Bonferroni method). Growth measured as reduction of redox dye measured by optical density at 590 nm minus optical density at 750 nm.

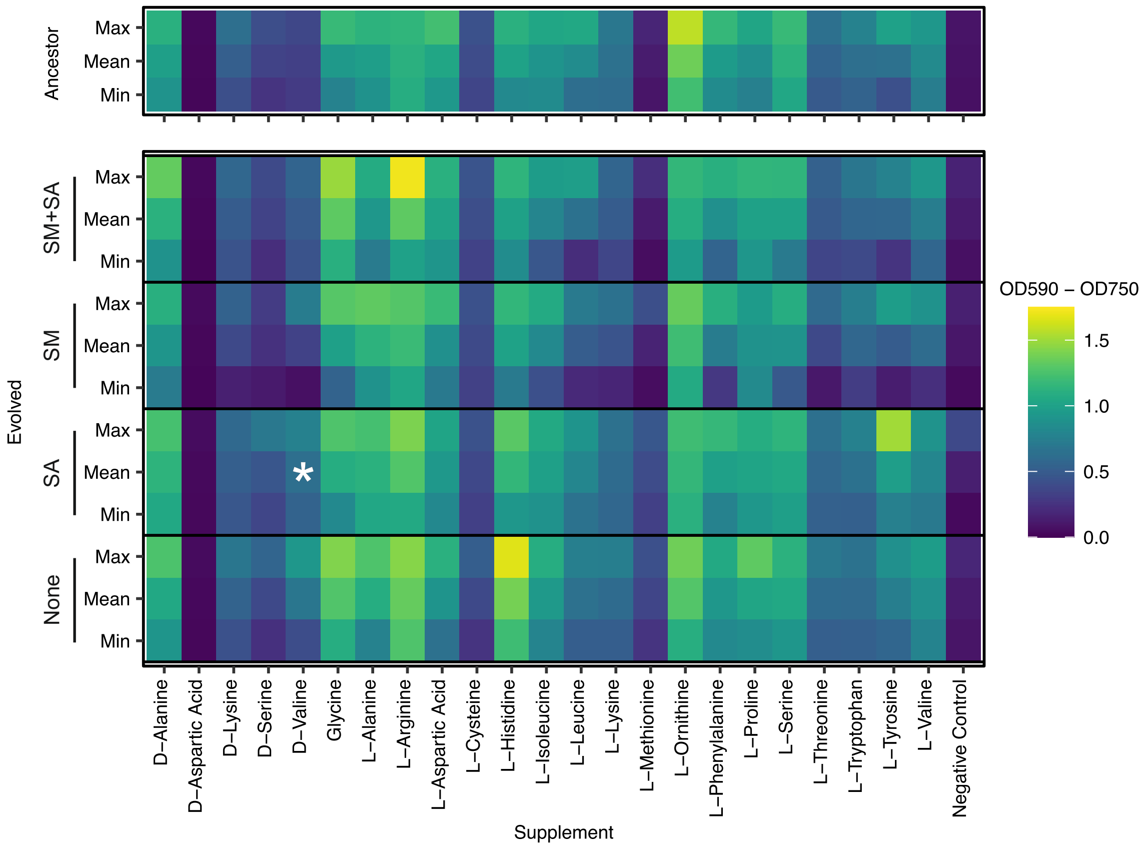


**Figure S8** Growth on sole nitrogen sources present in SCFM media. Mean, minimum and maximum growth of three replicates of ancestral *P. aeruginosa*, and mean, minimum and maximum growth of five colonies isolated from five different randomly selected populations evolved in the presence of mucin from each community. Stars show significant difference from ancestor (T-test, corrected for multiple testing using Holm–Bonferroni method). Growth measured as reduction of redox dye measured by optical density at 590 nm minus optical density at 750 nm.

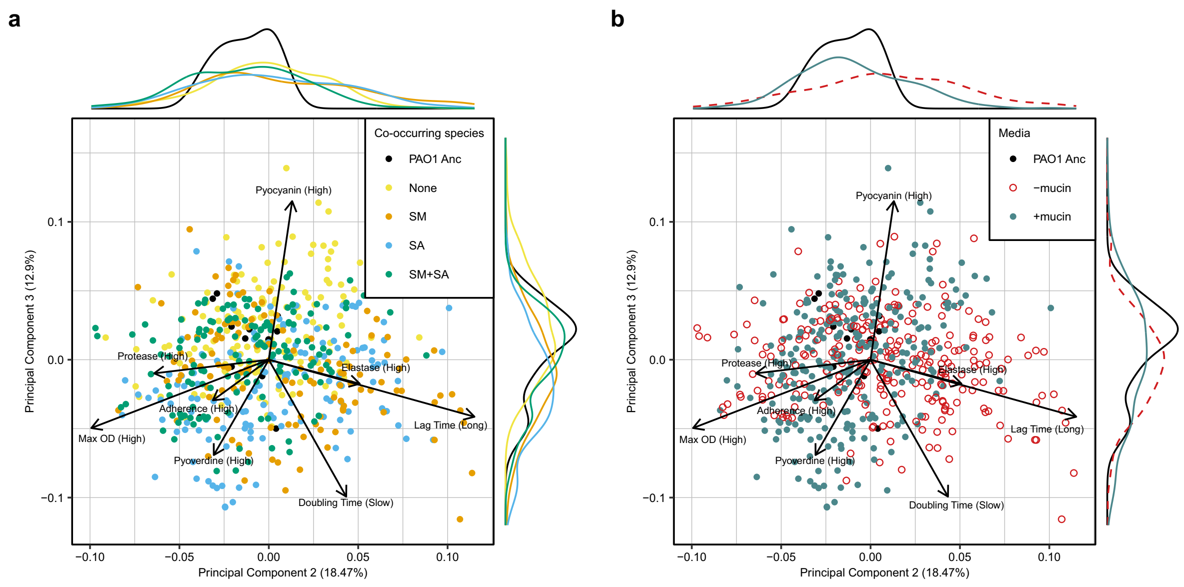


**Figure S9** Ordination plots from a principal component analysis of phenotypic trait measurements of ancestral strain (black points, n = 12) and evolved isolates (n = 72 per treatment). The second and third components describe 31.4% of the variance. The overlayed loadings plot (grey arrows) shows how strongly each phenotypic trait contributes to each principal component and the separation of the points. Points represent individual isolates, the density of points across each axis is shown at the top (PC2) and right (PC3) of the plots. **a** points coloured by co-occurring species identity, **b** points coloured by the presence of mucin.

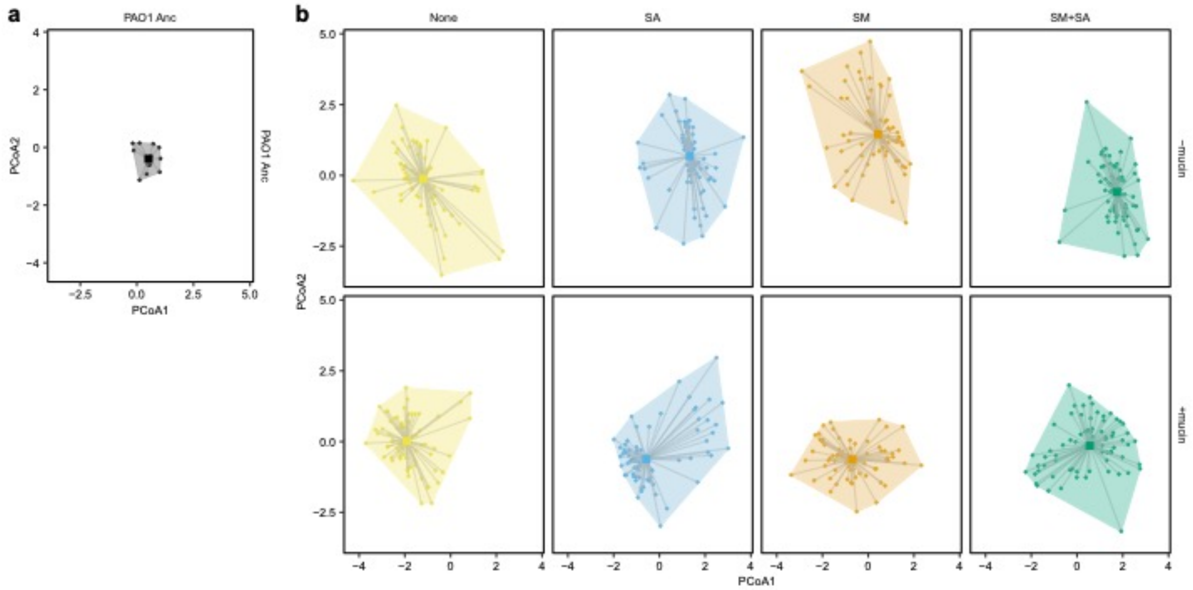


**Figure S10** Multivariate phenotypic dispersal (Beta-dispersal MDS plots) of **a** ancestor (n = 12), **b** within evolved treatments (n = 72 isolates per treatment) used to calculate the distance of each isolate to the treatment centroid presented in Figure 3a. The large square point shows the centroid of each treatment, and the points show the multivariate phenotype of each isolate. The grey lines represent the Euclidian distance of each isolate to the treatment centroid, and the hull encompassing all isolates represents the maximum diversification that occurred within treatment all isolates.

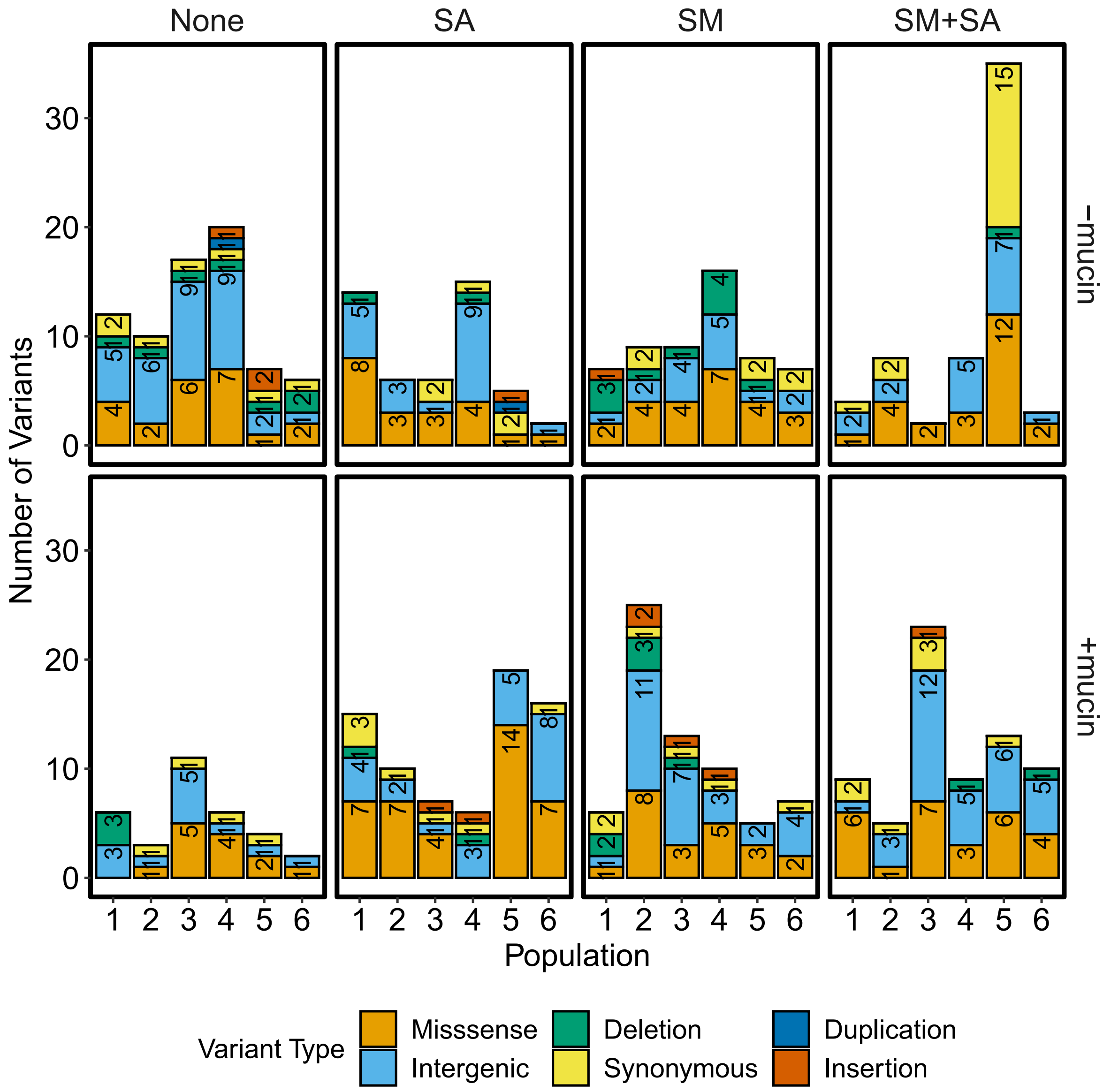


**Figure S11** Number of variants in each population broken down by mutation type. Faceted by co-occurring species identity and mucin treatment.

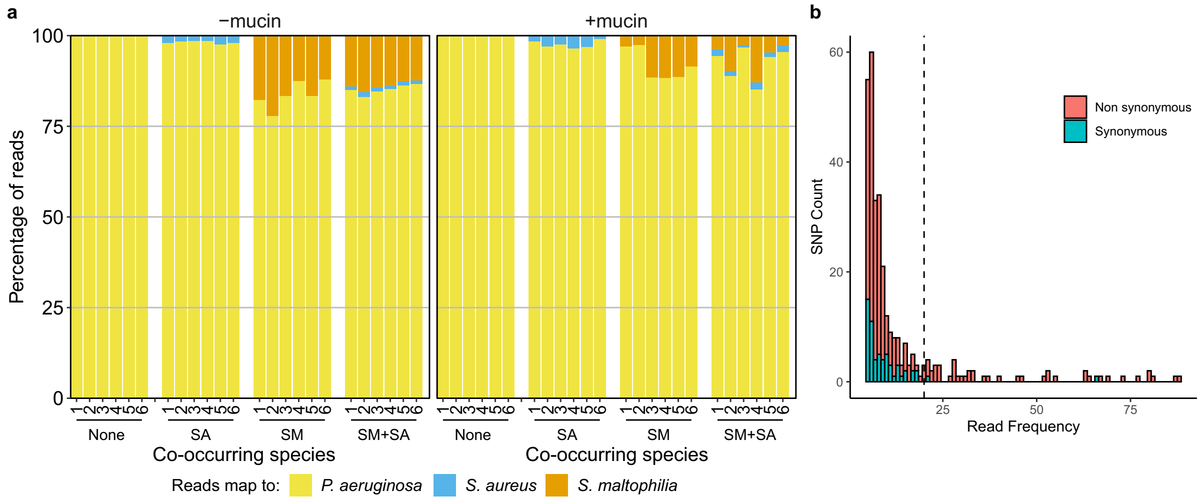


**Figure S12 a** Percentage of reads from each population mapped to each species. **b** Histogram showing frequency of all mutations within protein coding genes across all evolved populations, coloured by effect, synonymous vs non-synonymous. The vertical line at 20% frequency shows non-synonymous mutations that are likely under positive selection.

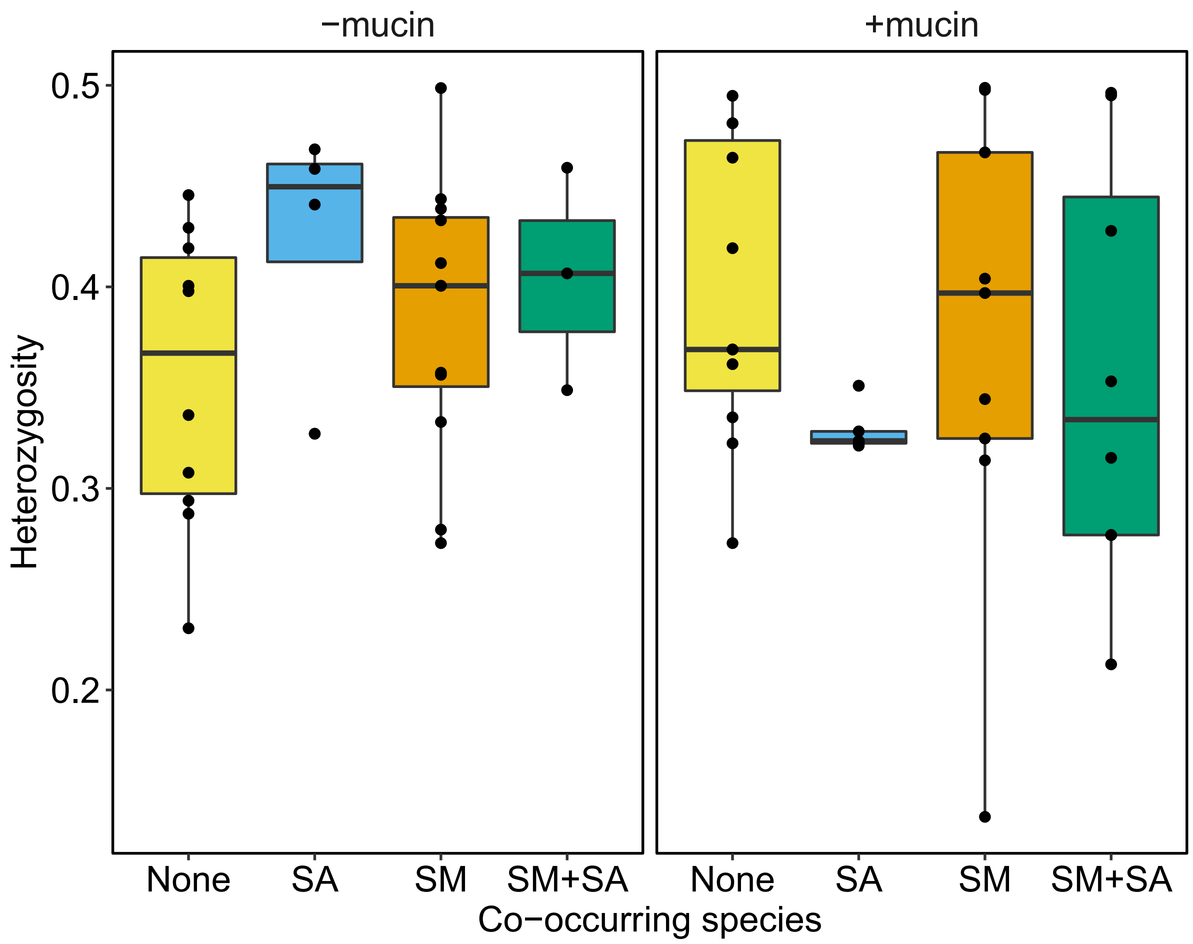


**Figure S13** Genetic diversity within populations based on frequencies of variants at all loci. Population heterozygosity was calculated as 2*pq*, where *p* is the allele frequency and $q = 1 - p$, excluding synonymous and low-frequency (<0.16) variants. Horizontal bars represent treatment median, the lower and upper hinges correspond to the first and third quartiles and the upper and lower whiskers extend to 1.5× interquartile range.

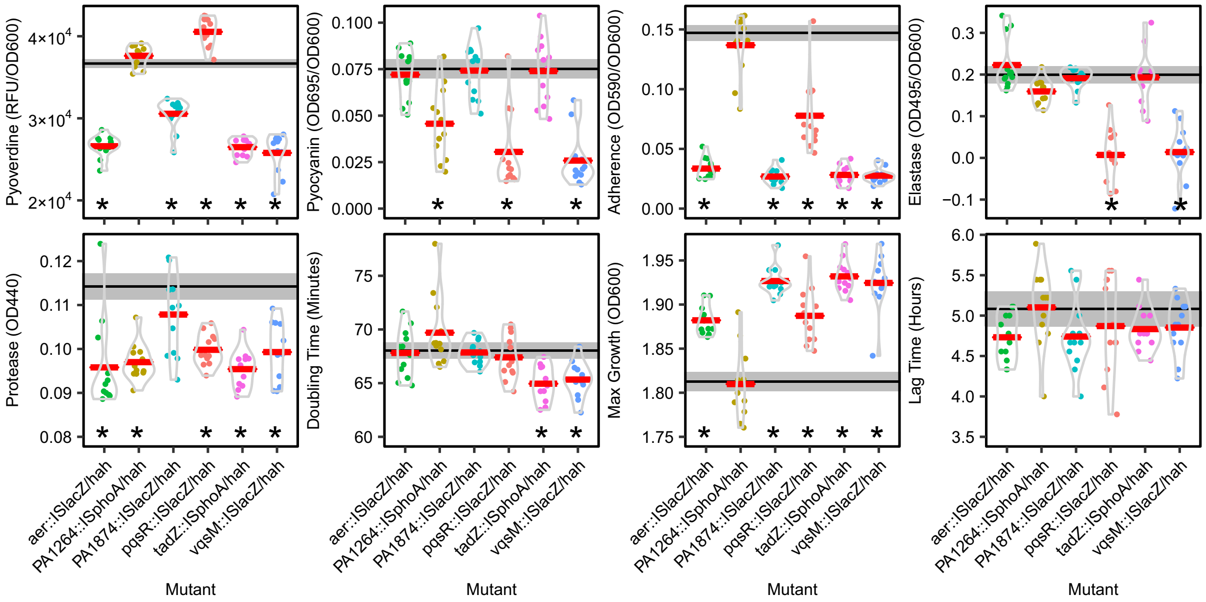


**Figure S14** Phenotyping of transposon mutants of the most common loci mutated in the evolved isolates. Points show phenotypes of 12 independent biological replicates per transposon mutant. The violin overlays show the distribution of the phenotypes within treatments and the red horizontal line shows the median across biological replicates (n = 12). The horizontal black line shows the mean phenotype of the parental strain (MPAO1), the grey shaded areas show the standard error of the mean (n = 12). Stars indicate significant difference between the transposon mutants and the parental strain (Wilcox Test, $p < 0.05$, corrected for multiple testing using Holm–Bonferroni method).

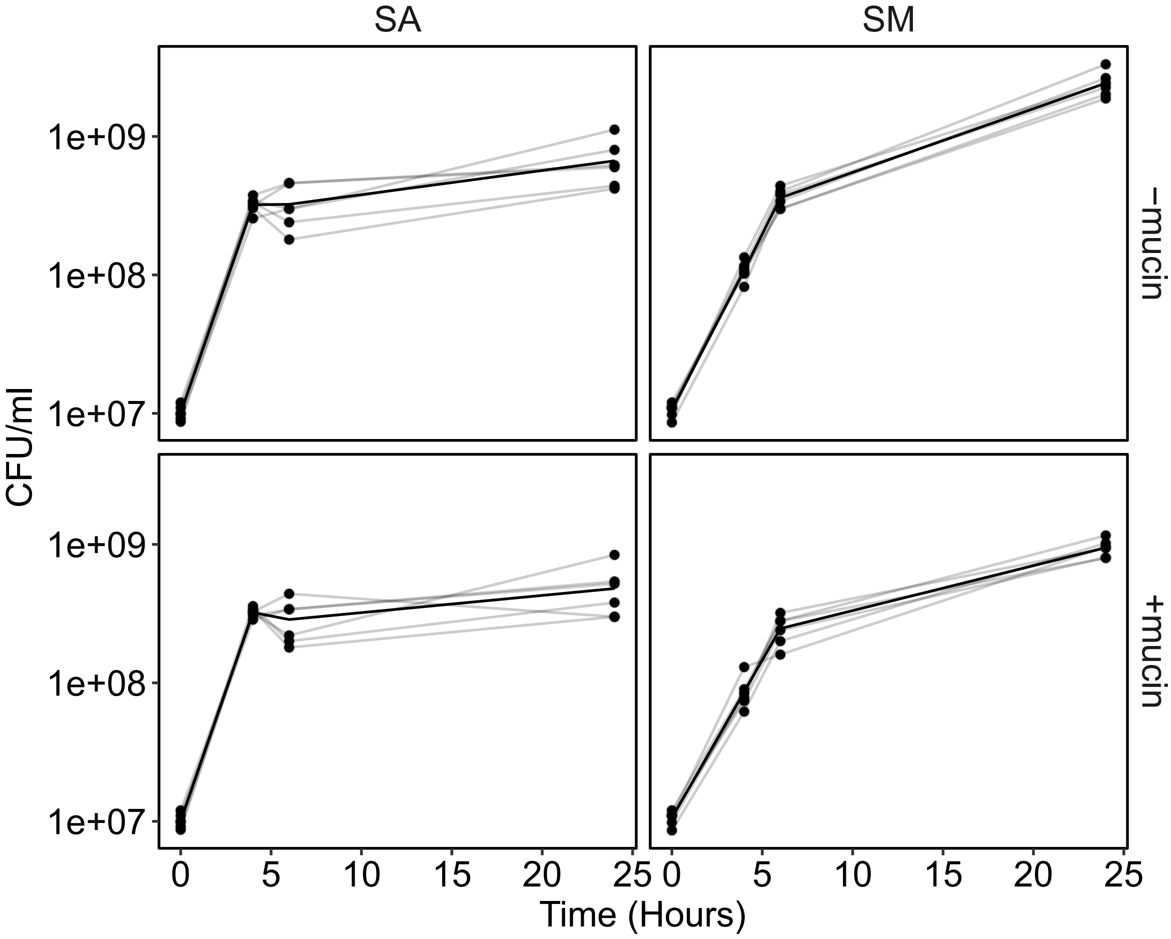


**Figure S15** Growth of *S. aureus* (SA) and *S. maltophilia* (SM) in SCFM with and without the supplementation of mucin. CFUs were measured at 0, 4, 6 and 24 hours post inoculation. The black line shows the mean of 6 independent replicates, represented by points.
